# Molecular Epidemiology of Hepatitis D Virus in the North-East Region of Romania

**DOI:** 10.3390/pathogens13090793

**Published:** 2024-09-13

**Authors:** Laura Iulia Grecu, Mariana Pavel-Tanasa, Lilia Matei, Camelia Sultana, Simona Maria Ruta, Razvan Ioan Grecu, Ramona Gabriela Ursu, Petru Cianga, Luminita Smaranda Iancu

**Affiliations:** 1 Department of Preventive Medicine and Interdisciplinarity Microbiology, Faculty of Medicine, “Grigore T. Popa” University of Medicine and Pharmacy, 700115 Iasi, Romania; lauraiuliagrecu@gmail.com (L.I.G.); grecuioanrazvan@gmail.com (R.I.G.); ramona.ursu@umfiasi.ro (R.G.U.); luminita.iancu@umfiasi.ro (L.S.I.); 2 Department of Emerging Viral Diseases, “Stefan S. Nicolau” Institute of Virology, 030304 Bucharest, Romania; madalina.sultana@umfcd.ro (C.S.); simona.ruta@umfcd.ro (S.M.R.); 3 Department of Immunology, Faculty of Medicine, “Grigore T. Popa” University of Medicine and Pharmacy, 700115 Iasi, Romania; petru.cianga@umfiasi.ro; 4 Laboratory of Immunology, St. Spiridon County Clinical Emergency Hospital, 700111 Iasi, Romania; 5 Department of Cellular and Molecular Pathology, “Stefan S. Nicolau” Institute of Virology, 030304 Bucharest, Romania; 6 Diaverum Romania, 011857 Bucharest, Romania; 7 Microbiology Department, Gynecology and Obstetrics Hospital-Cuza Voda, 700038 Iasi, Romania

**Keywords:** HDV sequencing, HDV-1 genotype, viral hepatitis, liver disease, epidemiology

## Abstract

The hepatitis D virus (HDV) superinfection of individuals with chronic hepatitis B virus (HBV) infection causes severe liver damage and the poorest long-term prognosis among viral hepatitis. This is attributed to the unique pathogenic mechanisms of HDV characterized by a direct cytopathic effect on hepatocytes and a significant impairment of the host immune response. The HDV genotype largely influences the extent of the pathogenic mechanisms with consequences on disease progression towards cirrhosis, liver decompensation, or hepatocellular carcinoma. In this context, identifying the circulating HDV genotypes in European regions with high prevalence, such as Romania, is crucial for effectively managing the long-term liver health. Here, we report the first comprehensive HDV study in Romania that clinically characterizes 82 patients and performs HDV genotyping by combining the nested-PCR reaction with sequencing analysis in 49 samples with an HDV-RNA load higher than 5000 IU/mL. While all isolates in our study belong to the HDV-1 genotype, the phylogenetic analysis based on sequence data from GenBank reveals the presence of the following potential three groups: (i) Italy and France; (ii) Spain; and (iii) Turkey, Iran, Pakistan, and Germany. This broad clustering highlights the recent surge in migration to and from Western Europe and the Middle East. Equally important, no differences in viral markers, clinical and paraclinical parameters, or treatment options were observed between these identified clusters. Nevertheless, this study considerably advances the understanding of hepatitis D epidemiology and clinical aspects in Romania.

## 1. Introduction

Hepatitis D virus (HDV) is a defective virus that uses the hepatitis B virus (HBV) envelope to infect hepatocytes, usually exacerbating the chronic infection with HBV [1]. Patients coinfected by HDV and HBV generally eradicate both pathogens, whereas chronic HBV carriers later infected by HDV develop chronic HDV infection and more severe liver damage [2]. On the same note, HDV superinfection has the poorest long-term prognosis of all viral hepatitis, causing cirrhosis in approximately 70% of cases in 5–10 years [3,4], followed by liver decompensation and hepatocellular carcinoma (HCC) in 3–15% of overall cases [5,6,7]. This can be explained by the particular pathogenic mechanisms of the virus having a direct cytopathic effect on hepatocytes or impairing the host immune response [8], or by its own genetic diversity with genotypes and subgenotypes that have different pathogenic characteristics and consequences on the evolution of liver disease [9].

The HDV genome is relatively simple and consists of a negative-sense single-stranded closed circular RNA, with a length between 1668 (HDV-1) and 1697 (HDV-5) nucleotides, having a content of GC pairs of approximately 60% (59.1–60.9%) regardless of genotype [9]. The differences between genotypes depend on nucleotide positions, representing less than 20% of the viral genome [10].

Currently, eight HDV genotypes (1–8) have been described, with specific geographic distribution: HDV-1 is ubiquitous, with the highest prevalence worldwide, especially present in Europe, the Middle East, North America, and North Africa; HDV-2 is more common in the Far East, Japan, Taiwan, and some parts of Russia [11]; genotype 3 is mainly found in the Amazon Basin, while genotype 4 was found largely in Taiwan and Japan; the other five to eight genotypes were detected in African patients who migrated to North Europe [12]. HDV-1 can be associated both with severe and mild forms of liver disease [13], while HDV-2 is related to moderate clinical symptoms [14]. HDV-3 causes a very aggressive clinical picture and the association with the F genotype of HBV caused fulminant acute hepatitis in most cases in South America [2]. Regarding HDV-4, there are studies that have shown that in some areas. this genotype was associated with a faster progression to liver cirrhosis [15]. On the other hand, there are areas such as Greece or the Far East where HDV infection evolves benignly, even without liver damage, which supports the idea that the circulating genotypes in different geographical regions contribute as risk factors in influencing the infection outcome [16].

Lately, the existence of genotypic subtypes has also been investigated and discussed. A recent European study indicated that the HDV genotype 1 could be classified into four subgenotypes (1a–1d), each corresponding to distinct geographical areas. In this study, it is also shown that genotypes 2 and 4 could each be subdivided into two subgenotypes. The accepted inter-genotypic differences are more than 10%, while the differences between the subgenotypes are between 3 and 10% [17]. HDV-1, which is globally prevalent, also shows the highest intragenotypic diversity (11.3–14.3%)—1a–1e. HDV-1a mainly originates from East Central Africa and Madagascar, while the HDV-1b subgenotype originates from other regions of Africa, such as Guinea [18]. HDV-1c has been reported especially in the Pacific Islands—Nauru and Kiribati—but also in Iran [19]. The HDV-1d subgenotype is the most widespread, being present on almost all continents: North Africa, the Middle East, Eastern and Western Europe, North America, and Asia [9]. HDV-1e is a relatively new identified subtype in sequences from France [20] and Italy [21]. Also in France, a new HDV-1 genotype was recently confirmed by sequencing using the long-read Oxford Nanopore Technologies coupled with a fully automated analysis pipeline [22]. Regarding the HDV genotypes 2 and 4, two subgenotypes were described for each genotype. For instance, subgenotype 2a has its origins in South Asia, while subgenotype 2b is mainly found in Yakutia—Eastern Siberia. Regarding HDV-4, subgenotype 4a was first described in Taiwan, and 4b in Japan [11]. The HDV-3 genotype has a localized distribution in South America and no distinct subgenotypes have yet been described; however, there is a group of strains reported to date divided into clusters: one cluster with origins in Brazil, Peru, and Venezuela and the other with origins in Bolivia [9]. HDV-5 to HDV-8 genotypes only came from patients in Africa, and it was noted that they could also be matched to two subgenotypes for each, with HDV-5 being the second most common genotype originating in sub-Saharan Africa. Interestingly, multiple studies have reported the detection of the HDV-5 genotype in Europe, linked to individuals of African descent in France [23], Italy [24], Germany [25], Spain [26], or the Netherlands [27]. HDV-7 originated in Cameroon, and HDV-6 and HDV-8 in Central Africa [28]. HDV-8 was also recently described in Brazil [9]. Using the genotypic and subgenotypic sequences for phylogenetic tree reconstruction, the origin of this virus in Africa was hypothesized, a theory also supported by the fact that there is a large proportion of genotypes originating in this continent that could be the center of diversification of HDV [9]. Intergenotypic diversity—the analysis of the HDV genome and proteins by sequencing—revealed a conservation of the virus structure and function, with minor changes at the molecular level [9]. Based on phylogenetic reconstruction analysis, the idea that this virus originates in Africa has been taken into consideration [9].

The most frequent genotype reported in Europe is HDV-1, followed by HDV-2 and African genotypes originating from immigrants coming from Africa [29]. The studies investigating the distribution of HDV-HBV double infection in Romania are scarce [30]; moreover, the studies that report the circulating HDV genotypes are insufficient and report only the presence of the HDV-1 genotype (Gheorghe L et al. [31] and Popescu GA et al. [32]). Other reports regarding the HDV genotypes present in the Romanian population are from international studies that analyzed isolated strains from immigrants of Romanian origin [23,33]. Importantly, the prevalence of HDV among HBV-infected subjects in Romania is relatively high, at 16.2% [30].

The aim of this study is to identify the circulating HDV genotypes in the population of northeastern Romania by sequencing, and to clinically characterize the patients included in this study for achieving a better understanding of the infection status in this area of Europe with a high prevalence.

## 2. Materials and Methods

### 2.1. Ethics

All the procedures were performed according to the General Code of Ethics in Scientific Research of Romania and this study was approved by the Institutional Ethics Committees—“Grigore T. Popa” University of Medicine and Pharmacy of Iasi and “St. Spiridon” University Hospital of Iasi, Romania, number 60750 (approval date: 13th of December 2018).

### 2.2. Study Population

A total of 82 HDV infected patients attending “St. Spiridon” Regional University Hospital in Iasi from January to July 2022, and participating in the National Screening Program for Viral Hepatitis LIVE (RO), were enrolled in this study. This screening program selected patients suitable for quantification of viremia in HCV-, HBV-, and HDV-infected patients after serological testing (anti-HCV Ab, HBsAg, total anti-HBc Ab, HBeAg, anti-HBe Ab, and anti-HDV Ab). Most patients had no prior history of clinical symptoms or a diagnosis of viral hepatitis infections.

**Figure 1:**
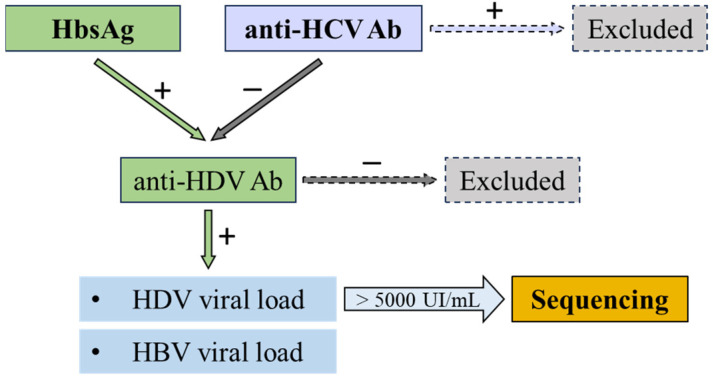
Protocol used for screening the patients with HDV infection.

The patients who tested positive for HBsAg and anti-HDV Ab were further included in our study for HDV sequencing and detection of both HBV and HDV viral loads. Patients who were also positive for HCV and HIV or those with autoimmune hepatitis or alcoholic liver disease were excluded (Figure 1).

Demographical data (date of birth, gender), paraclinical data (immunological markers such as HBsAg, total anti-HBc Ab, HBeAg, anti-HBe Ab, and anti-HDV Ab; biochemical markers such as alanine transaminase (ALT), aspartate aminotransferase (AST), gamma glutamyl transferase (GGT), total bilirubin, alpha fetoprotein, prothrombin time as international normalized ratio (INR), platelet blood count (PLT), and serum albumin level; HBV-DNA and HDV-RNA viral loads), and clinical data (cirrhosis diagnosis, decompensated cirrhosis, hepatocellular carcinoma diagnosis) were collected for each patient. Patients with incomplete paraclinical data were excluded from this study. Serum biomarkers for HCV and HIV infection were also recorded and patients with HCV or HIV infection were excluded.

### 2.3. Serological Testing

Serum samples from fresh venous blood were used for immunological markers testing. Qualitative commercial competitive enzyme immunoassay (DAB.CE from Dia.Pro, Milan, Italy) was used for anti-HDV Ab detection (both sensitivity and specificity > 98%). Also, quantification of HBsAg, anti-HBs Ab, HBeAg, and anti-HBe Ab was performed using chemiluminescent microparticle immunoassay method (CMIA) on the ARCHITECT System (Abbott, Green Oaks, IL, USA). The manufacturer’s instructions were followed for all the commercial kits. For the HBsAg test, the lower limit of linear quantification is 0.05 IU/mL. In the HBeAg test, an S/CO equal to or greater than 1.00 is considered reactive, while in the anti-HBe Ab test, an S/CO equal to or smaller than 1.00 is considered reactive.

### 2.4. Nucleic Acid Tests

All the samples with anti-HDV Ab positive results were included in this study and the HBV and HDV viral loads were tested. 

Nucleic acid extraction. DNA and RNA were extracted simultaneously from 1 mL of plasma using BioMagPure Extraction Kit (Biosan, Riga, Latvia) on BioMagPure System.

HBV-DNA quantification. After nucleic acid extraction, HBV-DNA viral load was tested using the HBV Real-TM Quant Dx assay (Sacace, Como, Italy). The analytical sensitivity or limit of detection (LOD) was 7 IU/mL with the 1.0 mL sample preparation procedure, and the specificity was 100%. The assay detected the HBV genotypes A, B, C, D, and H.

HDV-RNA quantification. The extracted RNA was reverse transcribed into cDNA and HDV-RNA viral load was tested using RoboGene HDV RNA Quantification Kit 2.0 with the first step of reverse transcription being included within the protocol and a LOD of 8 IU/mL (Roboscreen Diagnostics, Leipzig, Germany. The HDV-RNA levels measured using the RoboGene assay were converted to copies/mL by applying a conversion factor of 37, as previously reported [34,35]. The assay identifies all known HDV genotypes 1–8.

The manufacturer’s instructions were followed for all the commercial kits.

### 2.5. Sequencing Method

Extracted RNA samples with high viral loads were selected and stored at −80 °C until sequencing processing. A total of 49 samples with HDV-RNA viral loads higher than 5000 IU/mL were selected to be sequenced to ensure accurate detection and increase the chances of successfully implementing this method. The extracted RNA was first denatured at 95 °C for 5 min, then a nested-PCR was performed in order to obtain highly specific amplicons from conserved HDV genome, corresponding to a fragment of the HDAg gene (HDV genome M84917.1, nucleotides 883–1287) [32]. The average length of amplicons was around 404 bp to ensure adequate sequencing on ABI Prism 3130 Genetic Analyzer. First, a one-step reverse transcription polymerase chain reaction was performed (GoTaq^®^ probe 1-Step RT-qPCR Promega, Madison, Wisconsin, USA) using the outer primers forward 853IU and reverse 1302OD (Table 1). The reaction protocol was as follows: a first step at 45 °C for 15 min, followed by a second step at 95 °C for 2 min, followed by 40 cycles of 95 °C for 30 s, 60 °C for 30 s, 72 °C for 1 min, with a final step for elongation at 72 °C for 10 min. The amplicons obtained were introduced in a second PCR reaction (nested-PCR—DreamTaq Green PCR Master Mix Thermo Fisher Scientific, Waltham, Massachusetts, USA) using the inner primers forward HDV-E and reverse HDV-A detailed in Table 1.

In order to verify the results of the nested-PCR reaction, the amplicons were migrated in 2% agarose gel and examined under UV light. The samples with amplified DNA were purified from gel using Wizard^®^ SV Gel and PCR Clean-Up System (Promega, Madison, Wisconsin, USA). The purified DNA from the gel was used for sequencing with ABI Prism BigDye Terminator v3.1 Cycle Sequencing Kit (Applied Biosystems, Waltham, MA, USA) in an ABI Prism 3130 Genetic Analyzer.

### 2.6. Sequence Editing and Phylogenetic Analysis

Sequenced fragments were edited and assembled using DNADynamo software (#00011) and deposited into the GenBank database. Subsequently, the obtained sequences were aligned with the sequences of hepatitis delta virus (different genotypes) published in the GenBank (http://blast.ncbi.nlm.nih.gov/Blast.cgi (accessed on 5 November 2023)). Multiple alignments of sequences were performed using MUSCLE integrated in MEGA X software [40]. The evolutionary history was inferred using the maximum likelihood method and general time reversible model [41], and the robustness of the ML trees was inferred using 1000 bootstrap replicates [42]. Initial tree(s) for the heuristic search were obtained automatically by applying Neighbor-Join and BioNJ algorithms to a matrix of pairwise distances estimated using the maximum composite likelihood (MCL) approach, and then selecting the topology with a superior log likelihood value, a discrete Gamma distribution being used to model evolutionary rate differences among sites, with the rate variation model allowing for some sites to be evolutionarily invariable. Evolutionary analyses were conducted in MEGA X [43]. 

### 2.7. Statistical Analysis

IBM SPSS version 20 was used for data collection and analysis and statistically significant results were considered at a confidence level of at least 95%. The Shapiro–Wilk test and histogram were used to examine the normal distribution of data. For descriptive statistics, data with a normal distribution were represented as mean and standard deviation and non-parametric data as median and IQR. For comparative statistics, χ^2^ test, independent *t*-test, and non-parametric statistical tests, such as the Mann–Whitney U or Kruskal–Wallis tests, were used. To identify possible correlations between different demographic and paraclinical parameters, the Spearman correlation coefficient at a 95% confidence interval was used.

## 3. Results

### 3.1. Baseline Characteristics of the Patients in the Study

A total of 49 samples from patients included in this study were selected for sequencing. The mean age of the patients was 46 ± 14.4 years, and 55.1% were females. The representative biochemical parameters are presented in Table 2. The analysis of viral markers identified an increased mean value of HBsAg. Also, analysis of liver damage parameters data revealed elevated mean values of transaminases (ALT, AST, and GGT) and a low mean value of platelet count.

#### HBV and HDV Viremia

All patients included in this study had positive HDV-RNA viral loads higher than 5000 IU/mL with a median value of 961,210 IU/mL. All patients had positive HBV-DNA viral loads with a median value of 392 IU/mL (Table 3). The conversion from IU/mL to copies/mL is performed by applying a conversion factor of 37.

### 3.2. Clinical Aspects of Chronically Infected HDV Patients

At the time of inclusion in this study, seven (30.4%) of the patients with available clinical data (n = 23) already had a diagnosis of cirrhosis. Significantly higher HB antigen and INR values and significantly lower platelet counts (moderate thrombocytopenia) and albumin levels were observed in cirrhotic patients compared to non-cirrhotic ones (Table 4).

Other clinical findings regarding the degree of liver damage like the stage of fibrosis were not available, nor were data on liver disease decompensation events. There were also insufficient data regarding the presence/absence of a diagnosis of HCC.

#### Antiviral Therapy

**Figure 2:**
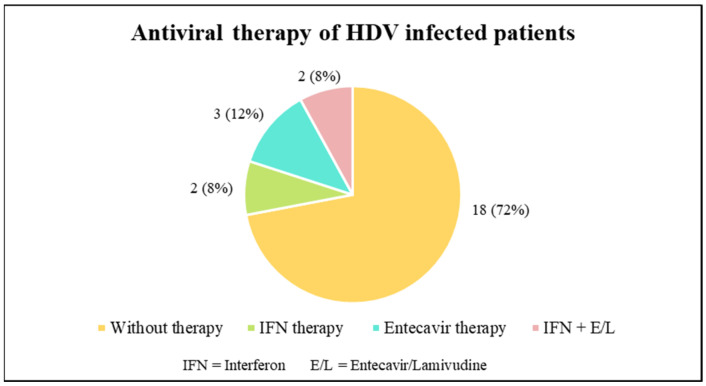
Types of antiviral treatment used for the patients included in this study.

Data about the antiviral treatment were available for only 25 patients. Of these, 18 were not treated, and the others were treated either with interferon or entecavir monotherapy, or a combination between interferon and entecavir or lamivudine (Figure 2).

There are no statistically significant differences between treatment groups in viral loads, viral markers, or paraclinical parameters (Kruskal–Wallis test). Also, no correlation was observed between the presence of treatment and the levels of viral loads, viral markers, or paraclinical parameters (Spearman test). 

Also, no correlation was noticed between treatment groups and the presence of cirrhosis (Chi square test). Neither the presence or absence of antiviral treatment was associated with the diagnosis of cirrhosis in our study cohort (Chi square test).

### 3.3. HDV Genotyping

#### 3.3.1. HDV Sequencing and Phylogenetic Analysis

**Figure 3:**
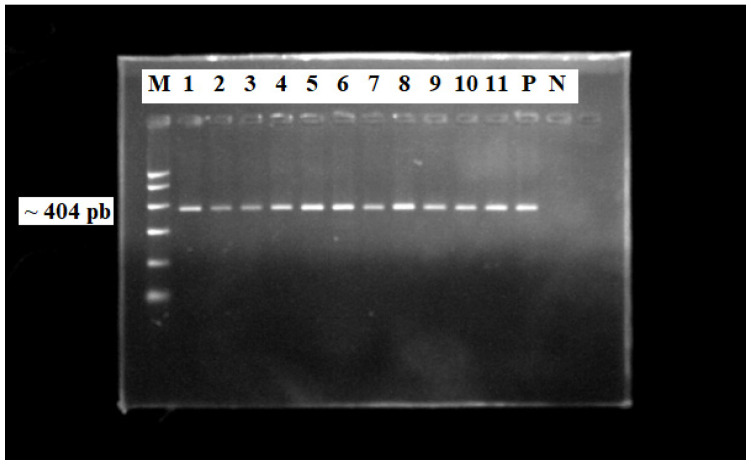
HDV amplicons obtained from nested-PCR and gel migration at 120 V in 2% agarose gel in TBE (M—molecular mass marker: Promega G316A; P—positive control; N—negative control) observed under UV light. Primers forward 853IU and reverse 1302OD in the first PCR reaction and forward HDV-E and reverse HDV-A in the second PCR reaction were used to obtain HDV genome-specific amplicons. These primers match reference sequences common to all HDV genotypes described to date.

To assign the genotype, we analyzed sequences encompassing the C-terminal half of HDAg, flanked by the highly conserved antigenomic cleavage domain and a short conserved region within the middle of the coding region which allowed this segment to be amplified even from widely differing isolates [36,44]. As such, we performed a nested PCR using primers for a highly conserved region of the HDV genome and the amplicons obtained in the final step were represented by a fragment of the HDAg gene (HDV genome M84917.1, nucleotides 883–1287) [45] with a medium size of 404 bp. The presence of the amplicons after the second PCR step was verified by migration in agarose gel and it was observed that only 44 out of 49 samples contained an amplified PCR product (Figure 3), amplicons that were included in the sequencing analysis.

**Figure 4:**
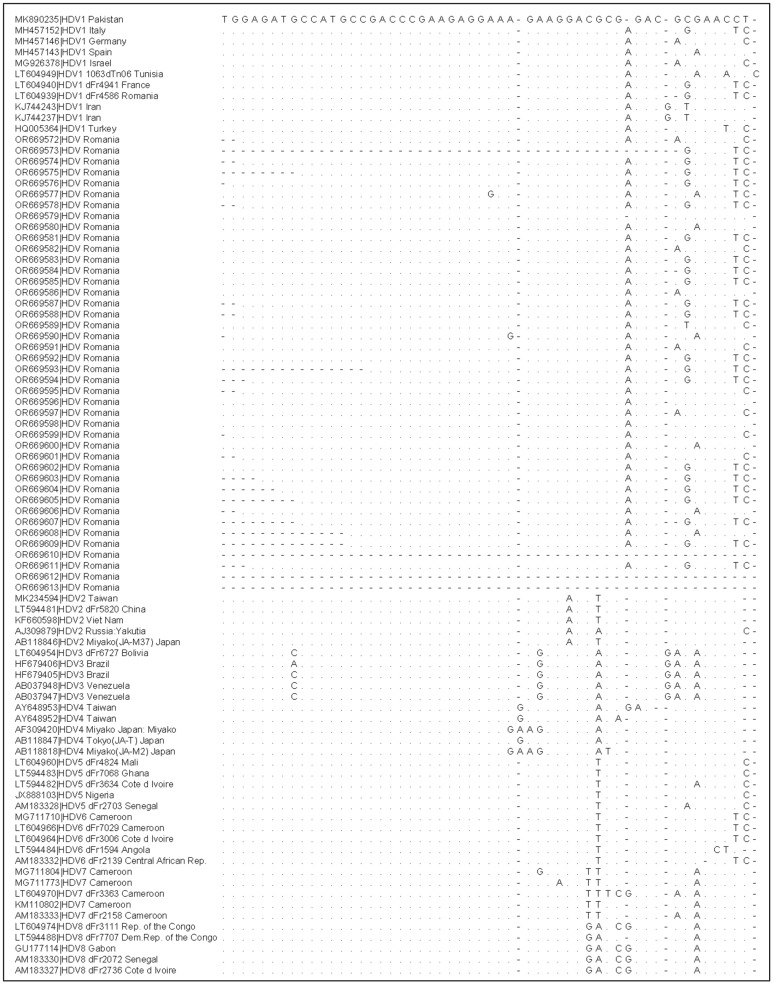
Sequence alignments with standard sequences from GenBank with the MUSCLE algorithm (for the entire alignment file, please see Supplementary Figure S1).

Next, only 42 samples containing the amplified PCR product for the specified fragment of the HDAg gene were successfully sequenced. The sequenced fragments of the viral genome were edited and assembled using the DNADynamo software, and then registered in the GenBank database under the accession numbers from OR669572.1 to OR669613.1. The fragment sequences were further analyzed by phylogenetic reconstruction using the alignment with standard sequences deposited in GenBank with the MUSCLE algorithm (Figure 4).

**Figure 5:**
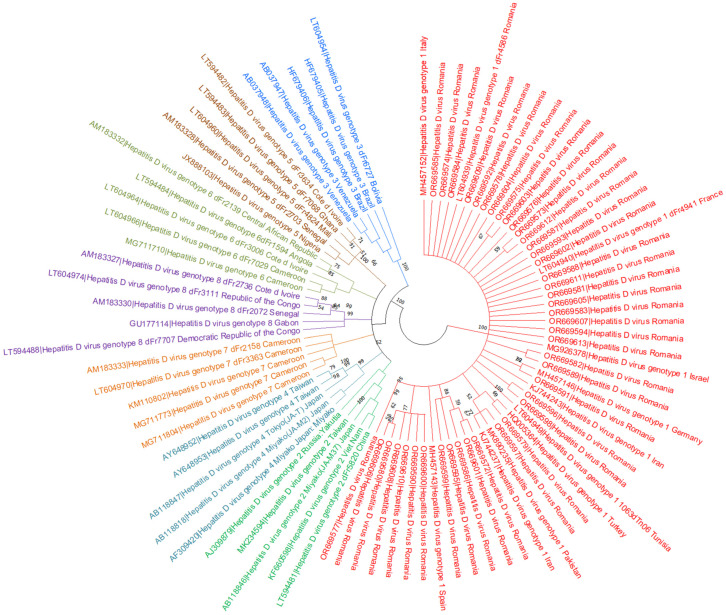
Phylogenetic relationships of HDV from Romania and other reported HDV sequences (different genotypes). The phylogenetic tree was inferred using the maximum likelihood method and the general time reversible model with 1000 bootstrap replication. The tree with the highest log likelihood (−6682.21) is shown. Bootstrap values are indicated at the nodes. The analysis involved 88 nucleotide sequences and there was a total of 419 positions in the final dataset. The scale bar is 0.10 nucleotide substitutions per nucleotide position.

It was found that all the isolates included in our study belong to the HDV-1 genotype (Figure 5). A nucleotide similarity that varies between 90% and 100%, depending on the first sequence considered as reference, was obtained between these isolates.

#### 3.3.2. Evolutionary Analysis—Phylogenetic Three Clustering

**Figure 6:**
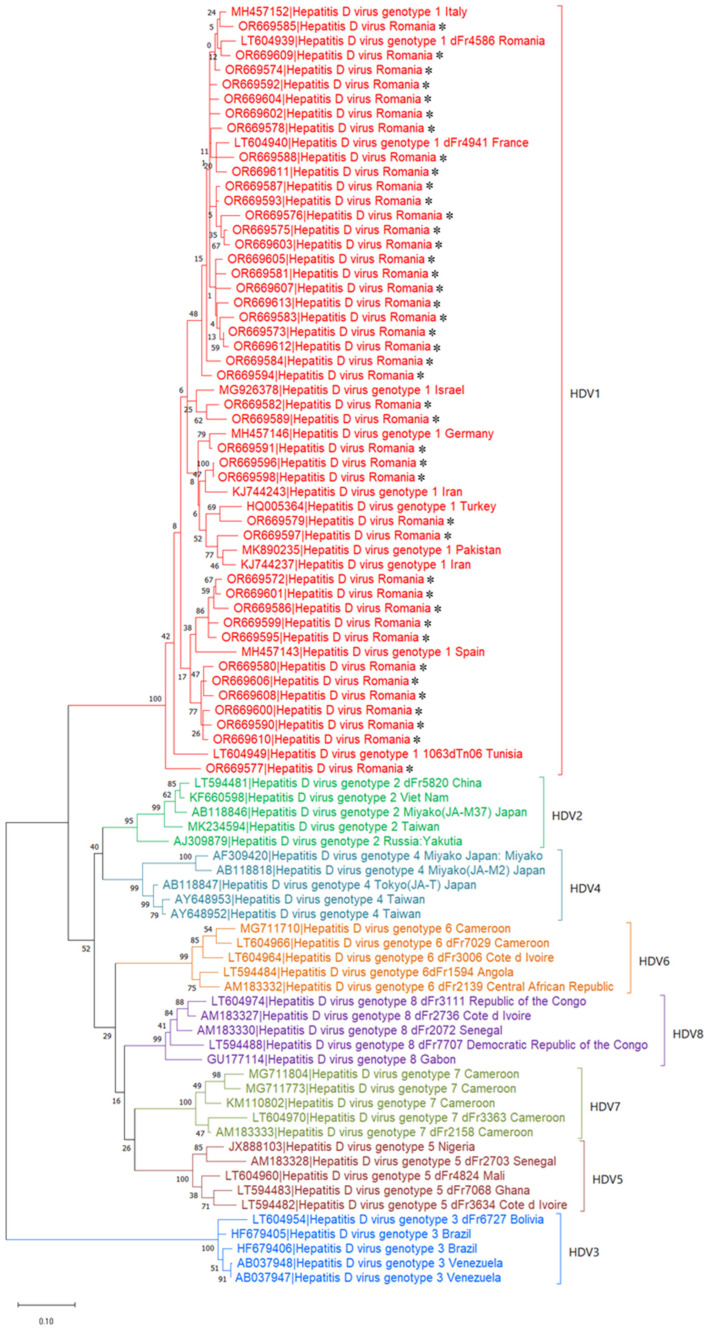
Phylogenetic relationships of HDV from Romania and other reported HDV sequences (different genotypes) and the grouping of the identified HDV-1 strains. The phylogenetic tree was inferred by using the maximum likelihood method and general time reversible model with 1000 bootstrap replication. The tree with the highest log likelihood (−6682.21) is shown. Bootstrap values are indicated at the nodes. The analysis involved 88 nucleotide sequences and there was a total of 419 positions in the final dataset. The scale bar indicates 0.10 nucleotide substitutions per nucleotide position. The HDV sequences detected in this study are marked with an asterisk (*).

**Figure 7:**
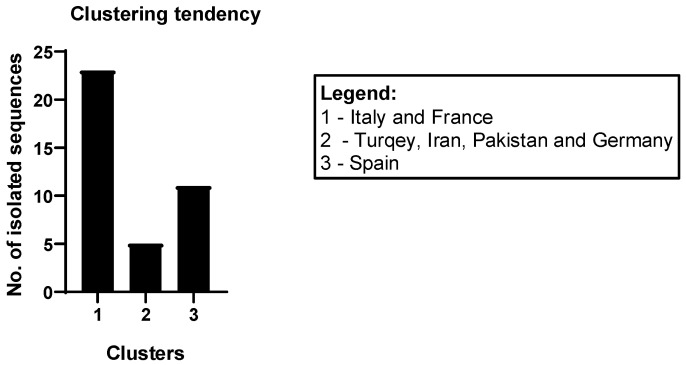
The clusters of HDV sequences from Romania isolated in this study—the origins of the sequences to which they are related.

Due to the lack of data, it was difficult to perform the HDV-1 subgenotyping. However, the phylogenetic tree built on the basis of isolates published in GenBank revealed a certain grouping tendency of the Romanian isolates. Three potential clusters seemed to emerge among the Romanian isolates: the first cluster is related to isolates from Italy and France; the second cluster is related to isolates originating from Turkey, Iran, Pakistan, and one from Germany; the third cluster is similar to isolates from Spain (Figure 6 and Figure 7). This broad clustering illustrates the intense migration phenomenon that has been taking place lately from and to Western Europe or the Middle East.

#### 3.3.3. Associations between Paraclinical and Clinical Data with Genotype

Next, we performed a comparative analysis of these sequence groups in relation to patients’ characteristics and no statistically significant differences were identified between clusters and demographics, levels of viral loads, viral markers, or paraclinical parameters (Kruskal–Wallis test). They were also not observed between the different sequence groups and the development of cirrhosis (Chi square test).

## 4. Discussion

Chronic delta virus infection remains a major health problem with a global impact despite the anti-HBV vaccine implementation. The incidence of HBV and further HDV infection has not yet declined sufficiently to be removed from the list of major health problems. A systematic review and meta-analysis, published in 2020 in collaboration with the WHO that included 282 studies with populations from 95 countries, evaluated the prevalence of HDV infection among those with HBsAg positive to be 4.5% (3.6–5.7 CI 95%) [46]. It has been estimated that approximately 12.0 (8.7–18.7 CI 95%) million people worldwide are positive for the anti-HDV antibody in the general population with active HDV infection or infection in the past [46].

The patients included in this study have an average age of 46 years, being a young population and with a slight predominance of the female gender. When comparing gender, advanced liver damage was observed to be associated with the male gender (as shown by the GGT level and HDV viremia), which would support other reports of poorer prognosis in HDV-infected male patients [47,48,49].

### 4.1. HDV-RNA and HBV-DNA Viremias

Only patients with HDV-RNA viral loads above 5000 IU/mL were included in this study, precisely to ensure that we had a sufficient amount of HDV genome to be further included in the sequencing analysis, in order to have a good quality of the isolated sequences. The median HDV viral load was around 960,000 IU/mL. On the other hand, the HBV-DNA viral load of these patients was not very high, the median value being around 392 IU/mL. From the comparison of the two viral levels, we can recall the notion of viral dominance of HDV introduced by Scharper et al. [50]; however, there are various patterns of HBV–HDV interaction. Numerous studies have described how HBV and HDV coexist and influence each other, and the statements were not always similar, eventually reaching the description of three patterns of coexistence of these two viruses: HDV dominance, HBV dominance, and a balance of HBV/HDV [50,51]. The factors that influence the existence of a certain pattern are still not fully elucidated.

### 4.2. Clinical Aspects of the Patients in Our Cohort

HDV infection presents the most aggressive evolution of liver disease of all hepatitis virus infections, being able to cause acute fulminant hepatitis or chronic hepatitis with rapid progression to cirrhosis, liver decompensation, or hepatocellular carcinoma. The presence of HDV increases the risk of decompensated cirrhosis by 2.2 times (CI 95% 0.8–5.7) in HBV-infected patients [30,52].

From a clinical point of view, we detected a percentage of 30% of the patients who have cirrhosis, but it is very important to note that there were patients with no clinical data who were included in this study. However, although a small sample of patients was studied, the specific characteristics for cirrhosis were highlighted by statistically significant differences in terms of slightly increased INR values, thrombocytopenia (even moderate in some cases), low albumin levels, but also higher levels of HBsAg in patients with a diagnosis of cirrhosis as compared to patients without a diagnosis of cirrhosis.

Regarding antiviral treatment, there was a percentage of 28% of patients in our cohort who followed at least one antiviral treatment regimen, but data were not available for all patients. The presence of a history of antiviral therapy was not associated with any level of paraclinical parameters, nor with the absence of cirrhosis.

### 4.3. HDV Genotyping

Considering that different HDV genotypes contribute to the ability to infect and spread in the hepatocyte and that different synthesis rates of the HD antigen can determine a certain rate of the new viral particle release, the pathogenicity of the virus largely depends on the genotype. For instance, HDV-8 showed the highest replication efficiency, while HDV-4 exhibited the lowest replication kinetics in the study of Wang et al. [53]. Considering the involvement of different genotypes in the clinical course of the patient with hepatitis D, an ideal approach to these patients should be completed with the determination of the HDV genotype. Therefore, among many viral and host factors, it has been observed that HDV genotypes may also contribute to the clinical course of liver disease. HDV-1 is associated with both severe and mild disease progression, and HDV-2 usually causes mild liver disease progression [54].

Different methods have been described over time to achieve this goal, from hybridization, restriction fragment length polymorphism (RT-LAMP), to RFLP and PCR followed by sequencing [55,56,57]. Among them all, genotyping by direct sequencing followed by phylogenetic analysis where specific sequences from each genotype are tracked, identified, and stored in different gene banks, e.g., GenBank, remains the gold standard used in the identification of the eight HDV genotypes. Very important in the use of such a method are the primers chosen, because they must allow the isolation of a region within the HDV genome that presents a high conservation of genetic material along the phylogenetic evolution, but also includes all the sought genotypes. Thus, in our study, primers were used that identified a sequence from the HDV genome of approximately 405 base pairs that codes for the HDV antigen [10,11]. Although only samples with high HDV viral loads were included in this study, for five patients out of forty-nine, no amplicons were obtained by the nested-PCR reaction.

In this study, HDV genotyping was successfully performed by direct sequencing of the amplicons obtained using the nested-PCR method. The amplicons obtained in our study were purified and further analyzed with the help of phylogeny studies using the maximum likelihood method and it was observed that all the sequences isolated belong to HDV 1 genotype. HDV-1 is ubiquitous, with a high prevalence worldwide, and is particularly present in Europe, the Middle East, North America, and North Africa [38]. This result confirms the previous observations which reported the HDV-1 genotype as having the highest prevalence in Romania and Eastern Europe [31,33]. 

Analysis of the HDV subgenotypes was difficult to achieve due to the low number of sequences corresponding to HDV-1 subgenotypes reported internationally in GenBank. However, after a careful analysis of the obtained phylogenetic tree, a grouping tendency of the isolates from our study was observed, a grouping that can possibly indicate the phenomenon of intense migration that has been taking place lately in Europe and beyond. Although this trend in clustering of isolated sequences is only informative, comparative analyses of the formed clusters and patient demographic, paraclinical, and clinical data were performed and no statistically significant differences were observed. The decline in genetic diversity of HDV in the new millennium may be a direct consequence of the implementation of the universal HepB birth dose vaccine since 1995, as well as the exertion of more effective screening methods in Romania.

According to the last EASL guidelines (August 2023), it was accepted that the HDV genotype has different effects on the course of the liver disease. The potential role of HDV-2 and HDV-5 in a benign course of the liver disease with a good virologic response to IFN-alpha treatment and a decrease in necro-inflammation was accepted. On the other hand, it was suggested that the HDV-1 and HDV-3 genotypes have a potential role in the worse prognosis of the infection with the development of cirrhosis and liver-related events, these types of patients presenting with persistent HDV viremia or high viral loads and increased necro-inflammation. It is strongly suggested that additional studies are needed to evaluate the influence of the HDV genotype and also the HBV genotype on the liver disease evolution independently of other host and environmental factors (such as ethnicity, gender, age, diabetes mellitus, obesity or alcohol consumption) in order to define the relevance in the clinical management of these types of patients [58].

HDV genotyping is important because it can be a diagnostic tool by which we can assess the patient’s evolution, estimate the response to treatment, and formulate a prognosis. Considering the involvement of different HDV genotypes in various clinical outcomes of hepatitis D infection, an ideal approach to these patients should be completed by the assessment of the HDV genotype. The limitations of this study were determined by the small number of samples, the incomplete data on clinical aspects and treatment, and the lack of information on the sequences of HDV-1 subgenotypes in the GenBank database. We believe that the sequencing results for the samples analyzed were not significantly affected by the limited sample size, but we cannot rule out the possibility that additional sequences could be detected if larger cohorts of patients were investigated. Expanding this study to include more patients could potentially uncover further diversity in HDV sequences and provide a more comprehensive understanding of the viral landscape. However, the comparison of HDV isolates between clusters may have been somewhat arbitrary due to the small number of patients. Selecting only samples with an HDV load higher than 5000 IU/mL for sequencing may have introduced bias in our study, as patients with lower viral loads might be infected with different HDV genotypes. This is important to address in future studies because, at least in theory, variations in replication efficiency are associated with different HDV genotypes, as previously mentioned [53]. Another limitation of our study is the focus on HDV genotyping without including HBV genotyping. In previous studies, we had identified that the most prevalent HBV genotype in our geographical area was D, followed by the A genotype. We also identified the presence of a double genotype (A + D), and surprisingly, we detected the F genotype in a very small number of patients [59,60,61]. However, the omission of HBV genotyping in this study may limit our understanding on the interaction between HDV and HBV genotypes, which could be important for analyzing disease progression and patient outcomes. Including HBV genotyping in future studies would provide a more comprehensive and clinically relevant perspective. Nevertheless, the previous data related to the HDV sequencing methods and results in Romanian population are scarce, and as a consequence, the present report can fill the gaps in the HDV infection picture in our country, being the first study in Romania from its North-East region that thoroughly addresses the molecular epidemiology providing a methodical examination of HDV sequencing data in relation to the patients’ clinical and paraclinical characteristics. The university hospital where this study was carried out is a regional hospital where patients from eight out of the forty-one counties of the country are diagnosed, treated, and monitored, a region covering about one quarter of the resident population. This study paves the way for more extensive research at a national level to investigate and compare the differences in the distribution and intensity of HDV circulation in Romania, a country marked by significant anthropological diversity across its various historical regions.

## 5. Conclusions

All the isolates from our study belong to the HDV-1 genotype, a result that confirms the previously reported HDV genotypes, and indicates a widespread genotype responsible for a wide variety of clinical presentations in HDV-infected patients. Also, we observed a clustering tendency of the analyzed isolates, which illustrates the phenomenon of intense migration that has occurred lately from and to Western Europe or the Middle East.

This study contributes to improving the current understanding of the epidemiology and clinical aspects of hepatitis D in Romania, emphasizing the importance of future research in this area. Moreover, it highlights the need for a better management of this infection, its treatment, and follow-up, but also the implementation of screening measures for chronic HBV carriers, through punctual public health actions and control measures in geographical areas with a large number of cases.

## Figures and Tables

**Table 1:** Primers used to perform nested-PCR.

PCR Step	Primers *		Sequence (5′–3′)	Position (nt)
First step PCR	853IU	Forward	CGGATGCCCAGGTCGGACC	853–871
	1302OD	Reverse	GGATTCACCGACAAGGAGAG	1322–1303
Second step PCR	HDV-E	Forward	GAGATGCCATGCCGACCCGAAGAG	887–910
	HDV-A	Reverse	GAAGGAAGGCCCTCGAGAACAAGA	1290–1267

* These primers had been used in previous studies for sequencing and phylogenetic reconstruction and allowed us to obtain comparable sequences and phylogenetic trees [36,37,38,39].

**Table 2:** Characteristics of patients included in this study.

Parameter		
	N (%)	
Gender (male/female)	22/27 (44.9%/55.1%)	
	**Mean**	**SD**
Age (years)	46.4	14.4
HBsAg (IU/mL)	16,871	10,693
PLT (×10⁹/L)	140.7	58.7
Albumin (g/dL)	33.8	22.1
ALP (U/L)	117.4	36.9
	**Median**	**IQR**
HBeAg (S/CO)	0.4	0.4–0.7
HBeAg, n (% of reactive)	11 (22.45%)	
Anti-HBe ab. (S/CO)	0.02	0.01–0.93
Anti-HBe Ab, n (% of reactive)	38 (77.55%)	
Anti-HBs Ab (IU/L)	0.6	0–1.5
ALT (U/L)	65.0	38.0–83.0
AST (U/L)	61.5	39.5–97.5
GGT (U/L)	50.0	32.0–87.0
AFP (ng/mL)	3.6	2.3–6.3
INR	1.1	1.07–1.25
Total protein (g/dL)	7.5	7.3–7.6

SD = standard deviation; IQR = interquartile range.

**Table 3:** Absolute and logarithmic (log_10_) HDV and HBV viremias—median values.

Viremia Type	Median	IQR
HDV-RNA (IU/mL)	961,210	123,577–20,588,441
HDV-RNA (log_10_)	5.9	5.03–7.3
HBV-DNA (IU/mL)	392.0	138.5–1151.0
HBV-DNA (log_10_)	2.6	1.1

IQR = interquartile range.

**Table 4:** Characteristics of chronically infected HDV patients with and without a diagnosis of cirrhosis—comparative analysis.

Parameters	Without Cirrhosis		Cirrhosis		*p*-Value
	N (%)		N (%)		
Gender (male/female)	7/9 (43.8%/56.2%)		4/3 (57.1%/42.9%)		0.554 *
	**Mean ± SD**		**Mean ± SD**		
Age (years)	45.0 ± 15.7		51.1 ± 13.4		0.355 ‡
HBsAg (IU/mL)	19,311.4 ± 9856.3		9598.9 ± 4507.4		**0.004 ‡**
PLT (×10⁹/L)	154.8 ± 52.3		86.0 ± 36.1		**0.002 ‡**
ALP (U/L)	110.0 ± 31.2		144.0 ± 46.4		0.112 ‡
	**Median**	**IQR**	**Median**	**IQR**	
HDV-RNA (IU/mL)	951572.0	196,068.0–8,995,024.3	61,812.0	46,896.0–747,912.0	0.285 †
HBV-DNA (IU/mL)	304.5	105.8–570.5	216.0	55.0–1243.0	0.789 †
HBeAg (S/co)	0.4	0.3–1.2	0.4	0.4–0.5	0.893 †
n (% of reactive)	4 (25%)		0 (0%)		0.273 *
Anti-Hbe Ab (S/CO)	0.02	0.01–0.7	0.09	0.02–1.5	0.228 †
n (% of reactive)	13 (81.25%)		5 (71.43%)		0.621 *
Anti-HBs Ab (IU/L)	0.6	0.0–1.3	0.1	0.0–1.1	0.456 †
ALT (U/L)	68.5	34.8–128.5	65.0	57.0–68.0	0.462 †
AST (U/L)	59.0	37.0–99.0	75.0	58.0–97.0	0.341 †
GGT (U/L)	44.5	26.8–93.8	68.0	36.0–114.0	0.548 †
AFP (ng/mL)	4.1	1.5–6.6	3.6	2.4–9.3	0.593 †
PT/ INR	1.1	1.0–1.2	1.4	1.2–1.6	**0.013 (Z = −2.48) †**
Total protein (g/dL)	7.6	7.4–7.9	7.4	7.1–7.7	0.229 †
Albumin (g/L)	43.9	33.0–59.7	30.7	25.7–51.6	**0.023 (Z = −2.27) †**

PLT = platelet count; ALP = alkaline phosphatase; ALT = alanine transaminase; AST = aspartate transaminase; GGT = gamma-glutamyl transferase; AFP = alpha fetoprotein; PT = prothrombin time; IQR = interquartile range. * Chi square test. ‡ *t* test—independent samples. † Mann–Whitney U test; *p*-values below 0.5 are highlighted with bold.

## Data Availability

The data are contained within the article.

## Supplementary Materials

The following supporting information can be downloaded at: https://www.mdpi.com/article/10.3390/pathogens13090793/s1, Figure S1: full sequence alignment file using standard sequences from GenBank and the MUSCLE algorithm.
