# Supplementary material for: Molecular Epidemiology of Hepatitis D Virus in the North-East Region of Romania
Source: Pathogens. 2024 Sep 13;13(9):793. doi: 10.3390/pathogens13090793 (PMC11435033; doi:10.3390/pathogens13090793)
Supplement: Supplementary file 1 [file pathogens-13-00793-s001.zip › pathogens-3148371-supplementary.pdf]

MK890235|HDV1 Pakistan	TGGAGATGCCATGCCGACCCGAAGAGGAAA-GAAGGACGCG-GAC-GCGAACCT-
MH457152|HDV1 Italy	..............................-..........A...-.G....TC-
MH457146|HDV1 Germany	..............................-..........A...-A......C-
MH457143|HDV1 Spain	..............................-..........A...-..A.....-
MG926378|HDV1 Israel	..............................-..........A...-A......C-
LT604949|HDV1 1063dTn06 Tunisia	..............................-..........A...-..A..A..C
LT604940|HDV1 dFr4941 France	..............................-..........A...-.G....TC-
LT604939|HDV1 dFr4586 Romania	..............................-..........A...--G....TC-
KJ744243|HDV1 Iran	..............................-..........A...G.T......-
KJ744237|HDV1 Iran	..............................-..........A...G.T......-
HQ005364|HDV1 Turkey	..............................-..........A...-.....T.C-
OR669572|HDV Romania	--............................-..........A...-A......C-
OR669573|HDV Romania	---G....TC-
OR669574|HDV Romania	--............................-..........A...-.G....TC-
OR669575|HDV Romania	--------......................-..........A...-.G....TC-
OR669576|HDV Romania	-.............................-..........A...-.G....TC-
OR669577|HDV Romania	...........................G..-..........A...-..A...TC-
OR669578|HDV Romania	--............................-..........A...-.G....TC-
OR669579|HDV Romania	..............................-..........-...-........-
OR669580|HDV Romania	..............................-..........A...-..A.....-
OR669581|HDV Romania	..............................-..........A...-.G....TC-
OR669582|HDV Romania	..............................-..........A...-A......C-
OR669583|HDV Romania	..............................-..........A...-.G....TC-
OR669584|HDV Romania	..............................-..........A...--G....TC-
OR669585|HDV Romania	..............................-..........A...-.G....TC-
OR669586|HDV Romania	..............................-..........A...-A.......-
OR669587|HDV Romania	--............................-..........A...-.G....TC-
OR669588|HDV Romania	--............................-..........A...-.G....TC-
OR669589|HDV Romania	..............................-..........A...-.T.....C-
OR669590|HDV Romania	-............................G-..........A...-..A.....-
OR669591|HDV Romania	..............................-..........A...-A......C-
OR669592|HDV Romania	..............................-..........A...-.G....TC-
OR669593|HDV Romania	---------------...............-..........A...-.G....TC-
OR669594|HDV Romania	---...........................-..........A...-.G....TC-
OR669595|HDV Romania	--............................-..........A...-.......C-
OR669596|HDV Romania	..............................-..........A...-........-
OR669597|HDV Romania	..............................-..........A...-A......C-
OR669598|HDV Romania	..............................-..........A...-........-
OR669599|HDV Romania	-.............................-..........A...-.......C-
OR669600|HDV Romania	..............................-..........A...-..A.....-
OR669601|HDV Romania	--............................-..........A...-.......C-
OR669602|HDV Romania	..............................-..........A...-.G....TC-
OR669603|HDV Romania	----..........................-..........A...-.G....TC-
OR669604|HDV Romania	------........................-..........A...-.G....TC-
OR669605|HDV Romania	--------......................-..........A...-.G....TC-
OR669606|HDV Romania	--............................-..........A...-..A.....-
OR669607|HDV Romania	--------......................-..........A...--G....TC-
OR669608|HDV Romania	-------------.................-..........A...-..A.....-
OR669609|HDV Romania	-------------.................-..........A...-.G....TC-
OR669610|HDV Romania	---
OR669611|HDV Romania	---...........................-..........A...-.G....TC-
OR669612|HDV Romania	---
OR669613|HDV Romania	---
MK234594|HDV2 Taiwan	..............................-....A..T..-...-.......--
LT594481|HDV2 dFr5820 China	..............................-....A..T..-...-.......--
KF660598|HDV2 Viet Nam	..............................-....A..T..-...-.......--
AJ309879|HDV2 Russia:Yakutia	..............................-....A..A..-...-.......C-
AB118846|HDV2 Miyako(JA-M37) Japan	..............................-....A..T..-...-.......--
LT604954|HDV3 dFr6727 Bolivia	.......C......................-.G.....A..-...GA.A....--
HF679406|HDV3 Brazil	.......A......................-.G.....A..-...GA.A....--
HF679405|HDV3 Brazil	.......C......................-.G.....A..-...GA.A....--
AB037948|HDV3 Venezuela	.......C......................-.G.....A..-...GA.A....--
AB037947|HDV3 Venezuela	.......C......................-.G.....A..-...GA.A....--
AY648953|HDV4 Taiwan	..............................G.......A..GA.--.......--
AY648952|HDV4 Taiwan	..............................G.......A.A-...-.......--
AF309420|HDV4 Miyako Japan: Miyako	.............................GAAG.....A..-...-.......--
AB118847|HDV4 Tokyo(JA-T) Japan	..............................G.......A..-...-.......--
AB118818|HDV4 Miyako(JA-M2) Japan	.............................GAAG.....AT.-...-.......--
LT604960|HDV5 dFr4824 Mali	..............................-.......T..-...-.......C-
LT594483|HDV5 dFr7068 Ghana	..............................-.......T..-...-.......C-
LT594482|HDV5 dFr3634 Cote d Ivoire	..............................-.......T..-...-..A....C-
JX888103|HDV5 Nigeria	..............................-.......T..-...-.......C-
AM183328|HDV5 dFr2703 Senegal	..............................-.......T..-...-.A.....C-
MG711710|HDV6 Cameroon	..............................-.......T..-...-......TC-
LT604966|HDV6 dFr7029 Cameroon	..............................-.......T..-...-......TC-
LT604964|HDV6 dFr3006 Cote d Ivoire	..............................-.......T..-...-......TC-
LT594484|HDV6 dFr1594 Angola	..............................-.......T..-...-....CT.--
AM183332|HDV6 dFr2139 Central African Rep.	..............................-.......T..-...-...-..TC-
MG711804|HDV7 Cameroon	..............................-.G....TT..-...-..A.....-
MG711773|HDV7 Cameroon	..............................-...A..TT..-...-..A.....-
LT604970|HDV7 dFr3363 Cameroon	..............................-......TTTCG...-A.A.....-
KM110802|HDV7 Cameroon	..............................-......TT..-...-..A.....-
AM183333|HDV7 dFr2158 Cameroon	..............................-......TT..-...-A.A.....-
LT604974|HDV8 dFr3111 Rep. of the Congo	..............................-......GA.CG...-..A.....-
LT594488|HDV8 dFr7707 Dem.Rep. of the Congo	..............................-......GA..-...-..A.....-
GU177114|HDV8 Gabon	..............................-......GA.CG...-..A.....-
AM183330|HDV8 dFr2072 Senegal	..............................-......GA.CG...-..A.....-
AM183327|HDV8 dFr2736 Cote d Ivoire	..............................-......GA.CG...-..A.....-

```
MK890235|HDV1 Pakistan                      GTGAGTGGAAACCCGC-TTTATTCACTGGGGTCGACAACTCTGAG----GAGAAA
MH457152|HDV1 Italy                         .......A........-..........................G.----....G.
MH457146|HDV1 Germany                       ................-..........................G.----......
MH457143|HDV1 Spain                         ................-....................G.....G.----......
MG926378|HDV1 Israel                        .......A.......--............................----....G.
LT604949|HDV1 1063dTn06 Tunisia             .......A.CC.....-.............TG...........G.----......
LT604940|HDV1 dFr4941 France                 .......A........-..........................G.----....G.
LT604939|HDV1 dFr4586 Romania                .......A........-..........................G.----....G.
KJ744243|HDV1 Iran                          ................-..........................G.----......
KJ744237|HDV1 Iran                          ................-............................----......
HQ005364|HDV1 Turkey                        ................-..........................G.----.T....
OR669572|HDV Romania                        ................-..........................G.----......
OR669573|HDV Romania                        .......A........-..........................G.----....G.
OR669574|HDV Romania                        .......A........-..........................G.----....G.
OR669575|HDV Romania                        .......A........-..........................G.----....G.
OR669576|HDV Romania                        ......AA........-..........................G.----....G.
OR669577|HDV Romania                        ................-.............T............G.----....G.
OR669578|HDV Romania                        .......A........-..........................G.----....G.
OR669579|HDV Romania                        ................-............................----....G.
OR669580|HDV Romania                        ................-..........................G.----....G.
OR669581|HDV Romania                        .......A........-..........................G.----....G.
OR669582|HDV Romania                        ................-............................----......
OR669583|HDV Romania                        .......A........-..........................G.----....G.
OR669584|HDV Romania                        .......A........-..........................G.----....G.
OR669585|HDV Romania                        .......A........-..........................G.----....G.
OR669586|HDV Romania                        ................-..........................G.----......
OR669587|HDV Romania                        .......A........-..........................G.----....G.
OR669588|HDV Romania                        .......A........-..........................G.----....G.
OR669589|HDV Romania                        ................-............................----......
OR669590|HDV Romania                        ................-..........................G.----....G.
OR669591|HDV Romania                        ................-..........................G.----......
OR669592|HDV Romania                        .......A........-..........................G.----....G.
OR669593|HDV Romania                        .......A........-..........................G.----....G.
OR669594|HDV Romania                        .......A........-..........................G.----......
OR669595|HDV Romania                        ................-..........................G.----....G.
OR669596|HDV Romania                        ................-..........................G.----......
OR669597|HDV Romania                        ................-............................----......
OR669598|HDV Romania                        ................-..........................G.----......
OR669599|HDV Romania                        ................-..........................G.----....G.
OR669600|HDV Romania                        ................-.............TG...........G.----....G.
OR669601|HDV Romania                        ................-..........................G.----......
OR669602|HDV Romania                        .......A........-..........................G.----....G.
OR669603|HDV Romania                        .......A........-..........................G.----....G.
OR669604|HDV Romania                        .......A....T...Y..........................G.----....G.
OR669605|HDV Romania                        .......A........-..........................G.----....G.
OR669606|HDV Romania                        ................-..........................G.----....G.
OR669607|HDV Romania                        .......A........-..........................G.----....G.
OR669608|HDV Romania                        ................-..........................G.----....G.
OR669609|HDV Romania                        .......A........-..........................G.----....G.
OR669610|HDV Romania                        --------------------------------...........G.----....G.
OR669611|HDV Romania                        .......A........-..........................G.----....G.
OR669612|HDV Romania                        -------------------------------------------------------
OR669613|HDV Romania                        -------------------------------------------------------
MK234594|HDV2 Taiwan                        ..........C..T.--A.CC..T.T.....AGT...-...GAG.----AGTGG.
LT594481|HDV2 dFr5820 China                 ..........C..T---G..C..T.T..A..GGT...-...GAG.----AGTGG.
KF660598|HDV2 Viet Nam                      ..........C..T---G..C..T.T.....GGT...-...GAG.----AGTGG.
AJ309879|HDV2 Russia:Yakutia                ..A.......C..T.--A.CC..T.T.....GGT...-...GAG.----AGTGG.
AB118846|HDV2 Miyako(JA-M37) Japan          ..........C..T---G..C..T.T.....AGT...-...GAG.----AGTGG.
LT604954|HDV3 dFr6727 Bolivia               ......-----T.TATTGCCC..T.T..TT.GGTG..-.C...G.ACCCAGT..T
HF679406|HDV3 Brazil                        ......-----T.TA.TGCCC..T.T..TT.GGTG..-.C...G.ACCCAGT..T
HF679405|HDV3 Brazil                        ......-----T.TA.TGCCC..T.T..TT.GGTG..-.C...G.ACCCAGT..T
AB037948|HDV3 Venezuela                     ..A...-----T.TA.TGCCC..T.T..TT.GGTG..-.C...G.ACCCAGT..T
AB037947|HDV3 Venezuela                     ..A...-----T.TA.TGCCC..T.T..TT.GGTG..-.C...G.ACCCAGT..T
AY648953|HDV4 Taiwan                        ..........C..TCG-A.CC..T.T.....GGT...-...GAG.----AGTGG.
AY648952|HDV4 Taiwan                        ..........C..TCG-A.CC..T.T.....GGT...-...GAG.----AGTGG.
AF309420|HDV4 Miyako Japan: Miyako          ..........C..TCG-A.CC..T.T.....GGT...-...GAG.----AGTGG.
AB118847|HDV4 Tokyo(JA-T) Japan             ..........C..TCG-A.CC..T.T.....GGT...-...GAG.----AGTGG.
AB118818|HDV4 Miyako(JA-M2) Japan           ..........C..TCG-A.CC..T.T.....GGT...-...GAG.----AGTGG.
LT604960|HDV5 dFr4824 Mali                  .....GT...C..TT.-A..C..T.T.....AGT...-...GAG.----AGTGG.
LT594483|HDV5 dFr7068 Ghana                 .....GT.....TTT--CAC...T.T.....AGT...-...GAG.----AGTGG.
LT594482|HDV5 dFr3634 Cote d Ivoire         .....GT.....TTT.--AC...T.T.....AGT...-...GAG.----AGTGG.
JX888103|HDV5 Nigeria                       ..........C.TT.--G.CT..T.T.....GGT...-...GAG.----AGTGG.
AM183328|HDV5 dFr2703 Senegal               ..........C.TTT--A.CC..T.T.....AGT...-...GAG.----AGTGG.
MG711710|HDV6 Cameroon                      .........C...TC--GC.C..T.T.....AGT...-...GAG.----AGTGG.
LT604966|HDV6 dFr7029 Cameroon              .........C...T-.-G..C..T.T.....AGT...-...GAG.----AGTGG.
LT604964|HDV6 dFr3006 Cote d Ivoire         .........C...TC--G..C..T.T.....AGT...-...GAG.----AGTGG.
LT594484|HDV6 dFr1594 Angola                .........C...TC--G..C..T.T.....RGTG..-...AAG.----AGTGG.
AM183332|HDV6 dFr2139 Central African Rep.  .........C...T-.-G..C..T.T.....GGTG..-....AG.----AGTGG.
MG711804|HDV7 Cameroon                      ..........GTTA---.C.C..T.T.....GGT...-...GAG.----AGTGG.
MG711773|HDV7 Cameroon                      ..........GTTA---.C.C..T.T.....GGT...-...GAG.----AGTGG.
LT604970|HDV7 dFr3363 Cameroon              ..........GTT.AA---.C..T.T.....GGT...-...GAG.----AGTGG.
KM110802|HDV7 Cameroon                      ..........GT.T---.C.C..T.T.....GGT...-...GAG.----AGTGG.
AM183333|HDV7 dFr2158 Cameroon              ..........GT.----...C..T.T.....GGT...-...GAG.----AGTGG.
LT604974|HDV8 dFr3111 Rep. of the Congo     ..........CT.T-.-A.CC..T.T.....GGT...-...GAG.----AGTGG.
LT594488|HDV8 dFr7707 Dem.Rep. of the Congo ..........CT.TT--.C.C..T.T.....GGT...-...GAG.----AGTGG.
GU177114|HDV8 Gabon                         ...........TTTCT-.C.C..T.T.....-GT...-...GAG.----AGTGG.
AM183330|HDV8 dFr2072 Senegal               ..........CT.T-.-..CT..T.T.....GGT...-...GAG.----AGTGG.
AM183327|HDV8 dFr2736 Cote d Ivoire         ..........CT.T-.-A.CC..T.T.....GGT...-...GAG.----AGTGG.
```

MK890235|HDV1 Pakistan	AGGGAGGATCGGATGGGAAGAGTATAT-CCTATGGGAATCCCCGGTCGCCCCTCG
MH457152|HDV1 Italy	............C..............-............T...TT......TA
MH457146|HDV1 Germany	....T.......C..........T...-............T..GAT......GA
MH457143|HDV1 Spain	............C...A..........-..............T..T..T...A
MG926378|HDV1 Israel	...........................-..........C..TA...TT.......A
LT604949|HDV1 1063dTn06 Tunisia	.......G...................-...C..........T..G.T.......A
LT604940|HDV1 dFr4941 France	............C..............-...C...............T.......A
LT604939|HDV1 dFr4586 Romania	............C..............-...C..........T...TT......TA
KJ744243|HDV1 Iran	...........................-..............T...TT.......A
KJ744237|HDV1 Iran	................A.......G..-..................TA.......
HQ005364|HDV1 Turkey	...........................-...C..........T..GTT......T.
OR669572|HDV Romania	............C...A..........-.......A...........T..T...A
OR669573|HDV Romania	............C..............-..............T...TT......TA
OR669574|HDV Romania	............C..............-..............T...TT......TA
OR669575|HDV Romania	............C.........C....-..............T...TT......TA
OR669576|HDV Romania	............C..............-...................T......TA
OR669577|HDV Romania	............CG..A.......A..-.....C........T.CGAT.......A
OR669578|HDV Romania	............C...........G..-..............A...TT......TA
OR669579|HDV Romania	............C..............-..............T..GT.........
OR669580|HDV Romania	.......G....C..............-...Y...............T.......A
OR669581|HDV Romania	............C..............-..............T...TT......TA
OR669582|HDV Romania	...........................-.....C........T...TT.......A
OR669583|HDV Romania	............C..............-..............T...TT......TA
OR669584|HDV Romania	............C..............-..............T...TT......TA
OR669585|HDV Romania	............C..............-..............T...TT......TA
OR669586|HDV Romania	....C.......C...A..........-...Y...............T..T...A
OR669587|HDV Romania	............C..............-..............T...TT......TA
OR669588|HDV Romania	............C..............-..............T...TT.......A
OR669589|HDV Romania	...........................-..............T...T........A
OR669590|HDV Romania	.......G....C..............-..............T..CTT......TA
OR669591|HDV Romania	............C..............-...Y..........T..YTT......TA
OR669592|HDV Romania	............C..............-..............T...TT......TA
OR669593|HDV Romania	............C..............-..............T...TT......TA
OR669594|HDV Romania	............C..............-.................GT.......TA
OR669595|HDV Romania	............C...A..........-..................TT.......A
OR669596|HDV Romania	...........................-..............T...TT.......A
OR669597|HDV Romania	........................A..-...Y...............A..T...A
OR669598|HDV Romania	...........................-..............T...TT.......A
OR669599|HDV Romania	............C...A..........-..............T...TT.......A
OR669600|HDV Romania	.......G....C..............-..............T..CTT......TA
OR669601|HDV Romania	............C...A..........-...................T..T...A
OR669602|HDV Romania	............C..............-..............T..CGT......TA
OR669603|HDV Romania	............C..............-..............T...TT......TA
OR669604|HDV Romania	............C..............-..............T...TT......TA
OR669605|HDV Romania	............C..............-..............T...TT......TA
OR669606|HDV Romania	.......G....C..............-...Y..........T...TT.......A
OR669607|HDV Romania	............C..............-..............T...TT......TA
OR669608|HDV Romania	.......G....C..............-...Y..........T...TT.......A
OR669609|HDV Romania	............C..............-..............T..CTT......TA
OR669610|HDV Romania	.......G....C..............-..............T...TT......TA
OR669611|HDV Romania	............C..............-...C...............T.......A
OR669612|HDV Romania	-...........C..............-..............T...TT......TA
OR669613|HDV Romania	------------C..............-..............T..YTT......TA
MK234594|HDV2 Taiwan	...CGTTG.T..GG....GAT.GGCT.A....C.........T.A.TT.....GA
LT594481|HDV2 dFr5820 China	...CGTTG.T..GG....GAT.GG.G.A..C.C.........T..GTT.....GA
KF660598|HDV2 Viet Nam	...CGTTG.T..GG....GAT.GG.G.A....C.........T...TT.....GA
AJ309879|HDV2 Russia:Yakutia	...CGCTGC.C.GG.....CC.G..T.A....C.........T..GTT..T..GA
AB118846|HDV2 Miyako(JA-M37) Japan	...CGTTG.T..GG....GAT.GGCG.A....C.........T...TT.....GA
LT604954|HDV3 dFr6727 Bolivia	GCCCG..GGGA.GC...GTA.ACCC..-A.C.......CTG.T..GTC..T.GGA
HF679406|HDV3 Brazil	GCCCG..GGGA.GC...GTA.ACCC..-A.........CTG.T..GTT..T.GGA
HF679405|HDV3 Brazil	.CCCG..GGG..GC...GTA.ACCC..-A.........CTG....GTT..TAGGA
AB037948|HDV3 Venezuela	.CCCG..GGGA.GC...GTA.ACCC..-A.........CTG.T..GTT..T.GGA
AB037947|HDV3 Venezuela	.CCCG..GGGA.GC...GTA.ACCC..-A.........CTG.T..GTT..T.GGA
AY648953|HDV4 Taiwan	...CG..GAG.AGG...CT...GGCTAA....C..A...T..T..A.T.....GA
AY648952|HDV4 Taiwan	...CG..GAG.AGG...CT...GGCTAA....C..A...T..T.AG.T.....GA
AF309420|HDV4 Miyako Japan: Miyako	...CG..GAGA.GG.C.CCC..GG.CAA....C..A......T.TGTT..T.GGA
AB118847|HDV4 Tokyo(JA-T) Japan	...CG..GAG.AGG...CT...GG.TAA....C..A...T..T.AGTT.....G.
AB118818|HDV4 Miyako(JA-M2) Japan	...CG..TAG..GG.ACCCC..GG.CAA....C..A......T.AGTT..T.GGA
LT604960|HDV5 dFr4824 Mali	...CG..GAG.CGG..ATCCC.GGCT.A..C.C.....CTG.T...TT.....TA
LT594483|HDV5 dFr7068 Ghana	...CG..GAG.CGG..ATCCC.GGCT.A..C.C.....CTG.T..GTC..T...A
LT594482|HDV5 dFr3634 Cote d Ivoire	...CG..GAG.TCG...TCCC.GG.T.A..C.C...T.CTG.T...TT..T..GA
JX888103|HDV5 Nigeria	...CG..GAG.CGG..TTCCC.GGCT.A....C.....CTG.T...TT.....TA
AM183328|HDV5 dFr2703 Senegal	...CG..GAG.SSG...KYCC.GGCT.A....C.....CTG.T...T......GA
MG711710|HDV6 Cameroon	...CG..GAG..CG....G....G.TAC..Y.C..A..CTG.T.AGWA.....GA
LT604966|HDV6 dFr7029 Cameroon	...CG..GAG..CG....G....G.TAC..C.C.....CTG.T.RGT......GA
LT604964|HDV6 dFr3006 Cote d Ivoire	...CG..GAG..CG....G....G.TAC..C....A..CTG.T...YA......A
LT594484|HDV6 dFr1594 Angola	...CG..GAG..CG....G..TYG.TAC..C....A...TG.T..GTT.....TA
AM183332|HDV6 dFr2139 Central African Rep.	...CG..GAGA.CG....G..CCG.TAC..C....A..CTG......T.....TA
MG711804|HDV7 Cameroon	...CG..GAG..GG..TCG..T.GGG.C..Y....A..CTG......T..T.GGA
MG711773|HDV7 Cameroon	...CG..GAGA.GG..TCG..C..GG.C..Y....A..CTG...A..T..T.GGA
LT604970|HDV7 dFr3363 Cameroon	...CG..GAG..GG..TCG..C..GGAC..C.......CTG.T...TT..T.GGA
KM110802|HDV7 Cameroon	...CG..GAG..GG..CCG.TC..GT.C.......A..CTG.T...TT..A.GGA
AM183333|HDV7 dFr2158 Cameroon	...CG..GAG..GG..TCG..C..GG.C..C.....M.CTG.T...TT..T.GGA
LT604974|HDV8 dFr3111 Rep. of the Congo	...CG..GAG..GG....CGT.GT.G.C..C....A...TG......T....GAA
LT594488|HDV8 dFr7707 Dem.Rep. of the Congo	...CG..GAG..GG....CGCTGC.G.C..C....AT.CTG......T.....GA
GU177114|HDV8 Gabon	...CG..GAG..GG..ATG...CT.G.C.......A..CTG.T...TT.....GA
AM183330|HDV8 dFr2072 Senegal	...CG..GAG..GG....CG.C.C.G.C.......A...TG.T...TT.....GA
AM183327|HDV8 dFr2736 Cote d Ivoire	...CG..GAG..GG....CGTTGT.G.C..C....A...TG.T....T....GGA

MK890235|HDV1 Pakistan	TGTCCAGCCCGTCCCCGGTCCGGTGGAAGGGGGACTCCGGGATTCCTTGCATGCT
MH457152|HDV1 Italy	..........C........T.T.GT...................CA.........T.
MH457146|HDV1 Germany	.......A..C..........T.GT.................C............
MH457143|HDV1 Spain	CA........C...........AGT...............A..............
MG926378|HDV1 Israel	C.........C..........T.GT.................CC...........
LT604949|HDV1 1063dTn06 Tunisia	C.........C..........TACT.................C............
LT604940|HDV1 dFr4941 France	G.........C..........T.GT..................G...........
LT604939|HDV1 dFr4586 Romania	..........C.....A....T.GA.................CA.T.........
KJ744243|HDV1 Iran	CA........C......TGT.T.GT.................CG.........T.
KJ744237|HDV1 Iran	.......................CT.................CC.........T.
HQ005364|HDV1 Turkey	..........C...........A...................C............
OR669572|HDV Romania	G.........C.....T.....AGT.................C..........T.
OR669573|HDV Romania	..........C.......A..T.GT......A..........CA...........
OR669574|HDV Romania	..........C..........T.GT.................CA...........
OR669575|HDV Romania	..........C..........T.GT......A..........CA...........
OR669576|HDV Romania	..........C.......A..T.GT...............A.CG.T.A.......
OR669577|HDV Romania	C.........C........T.T.GC.................C............
OR669578|HDV Romania	C.........C..........T.GC.................CA...........
OR669579|HDV Romania	..........C.....T.....A...........T....................
OR669580|HDV Romania	A......T..C...........AGTA................C........GA..
OR669581|HDV Romania	..........C..........T.GT.................CA...........
OR669582|HDV Romania	A.........C........T.T.GT.................CA...........
OR669583|HDV Romania	..........C.......ATT.T.GT.................CA...........
OR669584|HDV Romania	..........C........T.T.GT.................CA.........T.
OR669585|HDV Romania	.......T..C..........T.GA.................CA.T.A.......
OR669586|HDV Romania	C.........C.....A.....AGC.................C............
OR669587|HDV Romania	..........C........T.T.GT......A..........CA...........
OR669588|HDV Romania	C.........C..........T.GT...............A.CA...........
OR669589|HDV Romania	A.........C........T.T.GT...............A..G...........
OR669590|HDV Romania	.......T..C...........AGT.................C............
OR669591|HDV Romania	.......A..C..........T.GT.................C..........T.
OR669592|HDV Romania	..........C..........T.GA.................CG...........
OR669593|HDV Romania	..........C..........T.GT......A..........CA...........
OR669594|HDV Romania	..........C..........T.GT.................CG.......A...
OR669595|HDV Romania	G.........C.....A.....AGC.................C............
OR669596|HDV Romania	AA.....T..C..........T.GT...............A.CG.........T.
OR669597|HDV Romania	C...Y.....A...........AGC.................C..........T.
OR669598|HDV Romania	AA.....T..C..........T.GT...............A.CG.........T.
OR669599|HDV Romania	G......T..C.....A.....AGC.................C..........T.
OR669600|HDV Romania	.......T..C...........AGTA................C............
OR669601|HDV Romania	G.........C.....A.....AGY.................C..........T.
OR669602|HDV Romania	..........C..........T.GT.................CA...........
OR669603|HDV Romania	..........C.......A..T.GT......A..........CA...........
OR669604|HDV Romania	..........C......TG..T.GC.................C............
OR669605|HDV Romania	..........C..........T.GT..................G...........
OR669606|HDV Romania	A......T..C...........AGCA................C............
OR669607|HDV Romania	..........C..........T.GT.................CA...C.......
OR669608|HDV Romania	A......T..C...........AGT.................C...C........
OR669609|HDV Romania	..........CC.........T.GA.................CA.........T.
OR669610|HDV Romania	.......T..C...........AGTA........T.......CY...........
OR669611|HDV Romania	C.........C..........T.GT.................CA...........
OR669612|HDV Romania	..........C.......A..T.GT......A..........CA...........
OR669613|HDV Romania	..........C..........T.GT.................CA..CA.......
MK234594|HDV2 Taiwan	.......T..C.....C.....AGCA.....A.........A.C...C.C...CTG
LT594481|HDV2 dFr5820 China	.......T..C.....C.....AGC...............A.CA....C...CTG
KF660598|HDV2 Viet Nam	.......T..C.....C.....AGC...............A.CAT...C...T.G
AJ309879|HDV2 Russia:Yakutia	.......T..C..T..C.....AGT......A........A.C...AGT...TTG
AB118846|HDV2 Miyako(JA-M37) Japan	.......T..C.....C..T..AGA...............A.C.....C...CTG
LT604954|HDV3 dFr6727 Bolivia	....G.T...C..T..C..T...GAA................C...C....G...
HF679406|HDV3 Brazil	....G.T...C..T..C..T...GAA..............A.C...C....G.T.
HF679405|HDV3 Brazil	....G.T...C..T..C..T...GAA..............A.C...C....G...
AB037948|HDV3 Venezuela	....G.T...C..T..C..T...GAA..............A.C...C....G...
AB037947|HDV3 Venezuela	....G.T...C..T..C..T...GAA..............A.C...C....G...
AY648953|HDV4 Taiwan	.......G........TC....AGT......A........A.CGT.G........
AY648952|HDV4 Taiwan	C......G........TC....AGT......A...........GT.G........
AF309420|HDV4 Miyako Japan: Miyako	.......G........TC.T.TAGA......A........A.C.....C....G.
AB118847|HDV4 Tokyo(JA-T) Japan	.......G........TC....AGT......A.......................
AB118818|HDV4 Miyako(JA-M2) Japan	.......G........TC.T.TAGA......A........A.C.T...C....A.
LT604960|HDV5 dFr4824 Mali	.......G..C.....C.A..T.GA......A.....G....CATT.A....C.G
LT594483|HDV5 dFr7068 Ghana	C......T..C.....T....TAGA......A.....G....CG.TCA....CTG
LT594482|HDV5 dFr3634 Cote d Ivoire	.......T..C..T..C....T.GA......A.....G....CG...A....CTG
JX888103|HDV5 Nigeria	.......T..C..T..C....T.GT......A..T.....A.CGTT.A....TTG
AM183328|HDV5 dFr2703 Senegal	..........A..T..C....TAGA......A..T..G..A.CGTTCA....TTG
MG711710|HDV6 Cameroon	.......A........C.W....GA...T..A.....G..A.CCTG.A.....G.
LT604966|HDV6 dFr7029 Cameroon	C......G..A..T..C......GA...T..A..T..G..A.CAAGGA.....G.
LT604964|HDV6 dFr3006 Cote d Ivoire	C......A..T..T..C......GA...T..A..T..G..A.CGAGA......G.
LT594484|HDV6 dFr1594 Angola	C......T..T.....C.....AGA...T..A..T..G..A.CGTG.A.....G.
AM183332|HDV6 dFr2139 Central African Rep.	C......A..A.....C.A....GT...T..A..Y..G..A.CGTG.A.....G.
MG711804|HDV7 Cameroon	C...G.....T..T..C..T.T.GA.................CC...A...GTTG
MG711773|HDV7 Cameroon	....G.TG..C..T..C..T.T.GA.................C....A...GTTG
LT604970|HDV7 dFr3363 Cameroon	.......T..A.....C..T.T.GA...T..C..T.......C....A...GTTG
KM110802|HDV7 Cameroon	....G..T..C..T..C..T.T.GA.................CC...A...GTTG
AM183333|HDV7 dFr2158 Cameroon	Y......T........C..T.T.GA...C..C..........C....A...GTTG
LT604974|HDV8 dFr3111 Rep. of the Congo	.......T..A........T.T.GT...T..A........A..A..CA....C..
LT594488|HDV8 dFr7707 Dem.Rep. of the Congo	.......T...........T.T.GT...T.............CC...A....C..
GU177114|HDV8 Gabon	.......T..A......TGT.T.GT...T..A........A.CA..AA....C..
AM183330|HDV8 dFr2072 Senegal	C......T..A......TGT.T.GA...C..A........A.CA...A....C..
AM183327|HDV8 dFr2736 Cote d Ivoire	.......A..A......CGT.T.GT...T..A........A.CA...A....C..

```
MK890235|HDV1 Pakistan                        GGGGACGAAGCCGCCTCCGGGCGCTCCCCTCGATCCACCTTCGAGGGGGTTCACA
MH457152|HDV1 Italy                           ...............C.......................................
MH457146|HDV1 Germany                         ...............C..............................A.........
MH457143|HDV1 Spain                           ...............C..................G.T..................
MG926378|HDV1 Israel                          ...............C.......................................
LT604949|HDV1 1063dTn06 Tunisia               ...............C.......................................
LT604940|HDV1 dFr4941 France                  ...............C.......................................
LT604939|HDV1 dFr4586 Romania                 ...............C.......................................
KJ744243|HDV1 Iran                            ...............C.......................................
KJ744237|HDV1 Iran                            ................................G......................
HQ005364|HDV1 Turkey                          ...............C...............TG...G..................
OR669572|HDV Romania                          ...............C.......................................
OR669573|HDV Romania                          ...............C.......................................
OR669574|HDV Romania                          ...............C.......................................
OR669575|HDV Romania                          ...............C.......................................
OR669576|HDV Romania                          ...............C................G......................
OR669577|HDV Romania                          ...............C..............................A.........
OR669578|HDV Romania                          ...............C.......................................
OR669579|HDV Romania                          ...............C...............TG......................
OR669580|HDV Romania                          ...............C.......................................
OR669581|HDV Romania                          ...............C.......................................
OR669582|HDV Romania                          ......................................................T
OR669583|HDV Romania                          ...............C................G......................
OR669584|HDV Romania                          ...............C.......................................
OR669585|HDV Romania                          ...............C.......................................
OR669586|HDV Romania                          ...............C.......................................
OR669587|HDV Romania                          ...............C.......................................
OR669588|HDV Romania                          ...............C.......................................
OR669589|HDV Romania                          ...............C................G.....................T
OR669590|HDV Romania                          ...............C.......................................
OR669591|HDV Romania                          ...............C..............................A.........
OR669592|HDV Romania                          ...............C.......................................
OR669593|HDV Romania                          ...............C.......................................
OR669594|HDV Romania                          ...............C.......................................
OR669595|HDV Romania                          ...............C.......................................
OR669596|HDV Romania                          ...............C.......................................
OR669597|HDV Romania                          ..A............C................G......................
OR669598|HDV Romania                          ...............C.......................................
OR669599|HDV Romania                          ...............C.......................................
OR669600|HDV Romania                          ...............C...................................----
OR669601|HDV Romania                          ...............C.......................................
OR669602|HDV Romania                          ...............C.......................................
OR669603|HDV Romania                          ...............C...............TG......................
OR669604|HDV Romania                          ...............C.......................................
OR669605|HDV Romania                          ...............C.......................................
OR669606|HDV Romania                          ...............C.......................................
OR669607|HDV Romania                          ...............C.......................................
OR669608|HDV Romania                          ...............C................G......................
OR669609|HDV Romania                          ...............C.......................................
OR669610|HDV Romania                          ...............C................G......................
OR669611|HDV Romania                          ...............C.......................................
OR669612|HDV Romania                          ...............C.......................................
OR669613|HDV Romania                          ...............C.......................................
MK234594|HDV2 Taiwan                          A..............A................GA..T...C..GA..........
LT594481|HDV2 dFr5820 China                   A..............A................GA.TT...C..G..A........
KF660598|HDV2 Viet Nam                        A..............A................GA..T...C..G..A........
AJ309879|HDV2 Russia:Yakutia                  A........T.....C................GA.TT...C.AG.A.........
AB118846|HDV2 Miyako(JA-M37) Japan            A..............A................GA..T...C..GA.A........
LT604954|HDV3 dFr6727 Bolivia                 ...C........C..A................GCGGG..G..CGTT.........
HF679406|HDV3 Brazil                          ...C........C..A................GCGGG..G..C.TT.........
HF679405|HDV3 Brazil                          ...C....G...C..A................GCGGG..G..C.TT.........
AB037948|HDV3 Venezuela                       ............C..A................GCGGG..GK.C.TT.........
AB037947|HDV3 Venezuela                       ............C..A................GCGGG..G..C.TT.........
AY648953|HDV4 Taiwan                          .........C......................GA..T..G...............
AY648952|HDV4 Taiwan                          .........C......................GA..T..G...............
AF309420|HDV4 Miyako Japan: Miyako            T..............A................GAG.T..C...............
AB118847|HDV4 Tokyo(JA-T) Japan               .........C......................GA..T..G...............
AB118818|HDV4 Miyako(JA-M2) Japan             T..............A................GAGGT..C...............
LT604960|HDV5 dFr4824 Mali                    A.....A........C................GAGGTT.C...G.T.........
LT594483|HDV5 dFr7068 Ghana                   A.....A........C................GAAGT..AC..G.T.........
LT594482|HDV5 dFr3634 Cote d Ivoire           A.....R........C................GAGGTT.ACT.G.T.........
JX888103|HDV5 Nigeria                         A.....A........C................GAGATGT.C.AG.T.........
AM183328|HDV5 dFr2703 Senegal                 A...........A..C................GAGAGG.CC.AG.T.........
MG711710|HDV6 Cameroon                        ............C...................GA.TT..GC..G.T.........
LT604966|HDV6 dFr7029 Cameroon                .........A..C...................GA.TT......G.T.........
LT604964|HDV6 dFr3006 Cote d Ivoire           T...........C...................GA.TT..C...G.T.........
LT594484|HDV6 dFr1594 Angola                  T...........C....................A.TT......G.T.........
AM183332|HDV6 dFr2139 Central African Rep.    T...........C...................GA.TT...C..G.T.........
MG711804|HDV7 Cameroon                        A..A.....A.....C................G.TGT..C...G.A.........
MG711773|HDV7 Cameroon                        A..A...........C................G.GGT..C...G.A.........
LT604970|HDV7 dFr3363 Cameroon                A..............C................G.GGT...G..GA..........
KM110802|HDV7 Cameroon                        A..A...........C................G.TGT..C...............
AM183333|HDV7 dFr2158 Cameroon                A..A...........C................G.GGT..C.....A.........
LT604974|HDV8 dFr3111 Rep. of the Congo       A.....A..T.....C................GA.TT...C.AG.A.........
LT594488|HDV8 dFr7707 Dem.Rep. of the Congo   A...........T..C...............TGA.TT...C..G.A.........
GU177114|HDV8 Gabon                           A...........A..C..............T.GA.TT...C..G.A.........
AM183330|HDV8 dFr2072 Senegal                 A.....A..T.....C................GA.TT...C..G.A.........
AM183327|HDV8 dFr2736 Cote d Ivoire           A........T.....C................GA.TT...C..G.A.........
```

MK890235|HDV1 Pakistan	C C T C C A G T C G A C G G G C C G G C T A C T C T T C T T T C C C T T C T C T C G T C T T C C T C G G T C A
MH457152|HDV1 Italy	. . C . . . A C . . G . . . . G T . . . .
MH457146|HDV1 Germany	. . C . . . A C . . . .
MH457143|HDV1 Spain	. . C . . . A C . . G . . . . T . . . .
MG926378|HDV1 Israel	. . . . G . . . .
LT604949|HDV1 1063dTn06 Tunisia	. . . . G . . . . T . . . .
LT604940|HDV1 dFr4941 France	. . C . . . A C . . G . . . .
LT604939|HDV1 dFr4586 Romania	. . C . . . A C . . . . A . . . .
KJ744243|HDV1 Iran	. . . . G . . G . . . .
KJ744237|HDV1 Iran	. . . .
HQ005364|HDV1 Turkey	. . . . G . . . . T . . . .
OR669572|HDV Romania	. . C . . . A C . . . .
OR669573|HDV Romania	. . C . . . A C . . G . . . .
OR669574|HDV Romania	. . C . . . . C . . G . . . . T . . . .
OR669575|HDV Romania	. . C . . . A C . . . .
OR669576|HDV Romania	. . C . . . A C . . . . T . . . .
OR669577|HDV Romania	. . C . . . A C . C G . . . .
OR669578|HDV Romania	. . C . . . A C . T G . . . .
OR669579|HDV Romania	. . . . G . . . . C . .
OR669580|HDV Romania	. . . . . . A C . . G . . . .
OR669581|HDV Romania	. . C . . . A C . . . . T . . . .
OR669582|HDV Romania	. . C . . . A C . . . . T . . . .
OR669583|HDV Romania	. . C . . . . G . . . .
OR669584|HDV Romania	. . . . G T . . . .
OR669585|HDV Romania	. . C . . . A C . . G . . . . G T . . . .
OR669586|HDV Romania	. . C . . . A C . T G . . . . A . . . .
OR669587|HDV Romania	. . C . . . A C . . G . . . .
OR669588|HDV Romania	. . C . . . A C . . . . Y . . . .
OR669589|HDV Romania	. . C . . . A C . . G . . . . T . . . . C T . . . . .
OR669590|HDV Romania	. . . . . . A C . . G . . . .
OR669591|HDV Romania	. . . . Y . . . .
OR669592|HDV Romania	. . C . . . A C . . G . . . . T . . . . . Y . . . .
OR669593|HDV Romania	. . C . . . A C . . G . . . . G . . . .
OR669594|HDV Romania	. . . . . . A . . . G . . . .
OR669595|HDV Romania	T . C . . . .
OR669596|HDV Romania	. . . .
OR669597|HDV Romania	. . . . T . . . .
OR669598|HDV Romania	. . . .
OR669599|HDV Romania	. . C . . . A C . T G . . . . T . . . .
OR669600|HDV Romania	- -
OR669601|HDV Romania	. . C . . . A C . . . .
OR669602|HDV Romania	. . C . . . A C . T G . . . .
OR669603|HDV Romania	. . C . . . A C . . . .
OR669604|HDV Romania	. . C . . . A C . . G . . . .
OR669605|HDV Romania	. . C . . . A C . . . .
OR669606|HDV Romania	. . . . . . A C . . . .
OR669607|HDV Romania	. . . .
OR669608|HDV Romania	. . . . . . A C . . . . T . . . .
OR669609|HDV Romania	. . C . . . A C . . G . . . .
OR669610|HDV Romania	. . . . . . A . . . G . . . .
OR669611|HDV Romania	. . C . . . A C . T G . . . . A A . . . . . .
OR669612|HDV Romania	. . C . . . A C . T G . . . .
OR669613|HDV Romania	. . C . . . A C . . G . . . . R . . . .
MK234594|HDV2 Taiwan	T . C . . . . C . T G . . . . T . . . . T C . . . . A A . . . . . .
LT594481|HDV2 dFr5820 China	A . C . . . A C . C G . . . . T . . . . T C . . . . A A . . . . . .
KF660598|HDV2 Viet Nam	T . C . . . A C . C G . . . . T . . . . T C . . . . A A . . . . . .
AJ309879|HDV2 Russia:Yakutia	T . C . . . A C . C G . . . .
AB118846|HDV2 Miyako(JA-M37) Japan	T . C . . . A C . C G . . . . T . . . . T C . . . . A A . . . . . .
LT604954|HDV3 dFr6727 Bolivia	. . C . . . . G . C G . . . . G . . . . T . . . T C G . . . . . A . . . . T . . C T .
HF679406|HDV3 Brazil	. . C . . . . G . C G . . . . G T . . . . T . . . T C G . . . . . A . . . C T . . C . .
HF679405|HDV3 Brazil	. . C . . . . G . C G . . . . T . . . T C G . . . . . A . . . . T . . C . .
AB037948|HDV3 Venezuela	. . C . . . . G . C G . . . . T . . . T C G . . . . . A . . . C T . . C Y .
AB037947|HDV3 Venezuela	. . C . . . . G . C G . . . . T . . . T C G . . . . . A . . . C T . . C . .
AY648953|HDV4 Taiwan	T . C . . . A C . C G . . . .
AY648952|HDV4 Taiwan	T . C . . . A C . C G . . . .
AF309420|HDV4 Miyako Japan: Miyako	T . C . . . A C . C G . . . . T . . . . . T .
AB118847|HDV4 Tokyo(JA-T) Japan	T . C . . . A C . C G . . . .
AB118818|HDV4 Miyako(JA-M2) Japan	T . C . . . A C . C G . . . . T . . . .
LT604960|HDV5 dFr4824 Mali	T . C . . . A C . C G . . . .
LT594483|HDV5 dFr7068 Ghana	T . C . . . A C . C G . . . . C . . . .
LT594482|HDV5 dFr3634 Cote d Ivoire	T . C . . . A C . C G . . . .
JX888103|HDV5 Nigeria	T . C . . . A C . C G . . . .
AM183328|HDV5 dFr2703 Senegal	T . C . . . A C . C G . . . . G T . . . . T C . . . . T C T . . . . .
MG711710|HDV6 Cameroon	T . C . . . A C . C G . . . . T . . . . G . . . . C . . . . . .
LT604966|HDV6 dFr7029 Cameroon	T . C . . . A C . C G . . . . T . . . . T G . . . . C . . . . . .
LT604964|HDV6 dFr3006 Cote d Ivoire	T . C . . . A C . C G . . . . T . . . . T . . . . T G . . . . . . A . . . C T . . . . .
LT594484|HDV6 dFr1594 Angola	T . C . . . A C . C G . . . . T . . . . A . . . C . . . . . .
AM183332|HDV6 dFr2139 Central African Rep.	T . C . . . A C . C G . . . . T . . . . C . . . . . .
MG711804|HDV7 Cameroon	T . C . . . A C T C G . . . . G A T . . . . G . . . . . . A . . G A . . . . . .
MG711773|HDV7 Cameroon	T . C . . . A C T C G . . . . G . T . . . T G A . . . . . A . . G A . . . . . .
LT604970|HDV7 dFr3363 Cameroon	T . C . . . A C T C G . . . . G T . . . . G . T . . . T . . . . C . . G A . . . C . .
KM110802|HDV7 Cameroon	T . C . . . A C . C G . . . . G . . . . . T . T . . . . G . . . .
AM183333|HDV7 dFr2158 Cameroon	T . C . . . A C . C G . . . . T . . . . G T T . . . T . . . .
LT604974|HDV8 dFr3111 Rep. of the Congo	. . C . . . T C . C G . . . . T . . . . A . . . .
LT594488|HDV8 dFr7707 Dem.Rep. of the Congo	. . C . . . T C . T G . . . . T . . . .
GU177114|HDV8 Gabon	. . C . . . T C . T G . . . . C . A C . . . . T . . . . T . . . .
AM183330|HDV8 dFr2072 Senegal	. . C . . . T C . T G . . . .
AM183327|HDV8 dFr2736 Cote d Ivoire	. . C . . . T C . T G . . . . G . . . .

MK890235|HDV1 Pakistan	ACCTCTTGAGTTCCTCTTCTTCCTCCCTGCTGAGGCTCTTCCCTCCCGAGCTCAG
MH457152|HDV1 Italy	.....CGA...........C......T....C...TG...........C......
MH457146|HDV1 Germany	.......A..........................CG............C......
MH457143|HDV1 Spain	....G......CCCTC...
MG926378|HDV1 Israel	.....CGA...........C......T...................TCC.GC...
LT604949|HDV1 1063dTn06 Tunisia	....TC...CC.GC...
LT604940|HDV1 dFr4941 France	.....CGA...........C......T....C................C.G....
LT604939|HDV1 dFr4586 Romania	..T..CGA...........C..T........C...T............C.GA...
KJ744243|HDV1 Iran	....TC....................T........T...........CC.GA...
KJ744237|HDV1 Iran	.......A...........................T....T.......C......
HQ005364|HDV1 Turkey	....T.............................CT..........TCC.GCT..
OR669572|HDV Romania	.....CGA...........C......T.............G........CGAT..
OR669573|HDV Romania	.......A...........C..---------------------------------
OR669574|HDV Romania	.....CGA..................T....C...T............C.....A
OR669575|HDV Romania	...................C..T........C...TG...........C.....A
OR669576|HDV Romania	.......A...........C..G........C...T............C.T...A
OR669577|HDV Romania	....TC.............C....................T..C...TG.GAG..
OR669578|HDV Romania	.......A........A.TC......T....C................C.....A
OR669579|HDV Romania	G....CCA..........................CT.............------
OR669580|HDV Romania	.......A...........C......T.............T..C....TCGAT..
OR669581|HDV Romania	.......A...........C..T........C...T............C.GC...
OR669582|HDV Romania	.......A..................T...................TCC.GC...
OR669583|HDV Romania	.......A...........C...........C...T..............GC...
OR669584|HDV Romania	.......A.......................C................C......
OR669585|HDV Romania	.....CGA..........TC......T....C................C.GC...
OR669586|HDV Romania	..T..CGA...........C......T.............G........CGA...
OR669587|HDV Romania	.......A...........C..G........C................C.T...A
OR669588|HDV Romania	..............C................C..............T.C.GC...
OR669589|HDV Romania	......C...................T...................TCC.GCT..
OR669590|HDV Romania	..T....A..C........C....................T..C.....CGAT..
OR669591|HDV Romania	.....C............................CT............C......
OR669592|HDV Romania	..Y..CGA...........C......T....C................C......
OR669593|HDV Romania	.......A...........C..G........C................C......
OR669594|HDV Romania	...................C......T....C................C......
OR669595|HDV Romania	.....CGA...........C......T.............G........CGA...
OR669596|HDV Romania	.......A..........................CT............C.GAT..
OR669597|HDV Romania	.......A...........................T..........T........
OR669598|HDV Romania	.......A..........................CT............C.GAT..
OR669599|HDV Romania	.....CGA...........C......Y.............G........CGAT..
OR669600|HDV Romania	---
OR669601|HDV Romania	.....C.A..........TC..T...T.............G........CGA...
OR669602|HDV Romania	.......A..............G...T....C...T............C......
OR669603|HDV Romania	......................T........C...T............C.....A
OR669604|HDV Romania	.......A...........C..G...T....C................C......
OR669605|HDV Romania	.......A...........C..W...T....C................C.GA...
OR669606|HDV Romania	.......R...........C......T.............T..C.....CGAT..
OR669607|HDV Romania	...................C......T....C...GA.............GC...
OR669608|HDV Romania	.......A...........................Y....T..C.....CGAT..
OR669609|HDV Romania	.....GAA...........C......T....C...T............C.GC...
OR669610|HDV Romania	.......R...........C......T.............T..C.....CGAT..
OR669611|HDV Romania	.....--
OR669612|HDV Romania	.......A...........C..G........C................C.G....
OR669613|HDV Romania	.......A...........C......T....C...A............C.GA...
MK234594|HDV2 Taiwan	....TCC............C....................T.......C.GAG..
LT594481|HDV2 dFr5820 China	....TC.............................T....T.........GAG..
KF660598|HDV2 Viet Nam	.....C.............................T....T.........GAG..
AJ309879|HDV2 Russia:Yakutia	.....C.............................T....T.......C.GAG..
AB118846|HDV2 Miyako(JA-M37) Japan	.....C...........................A......T.........GAG..
LT604954|HDV3 dFr6727 Bolivia	...G..CA........C........T.G.......TG...........C.GC...
HF679406|HDV3 Brazil	G....CG..................TTG.......TG...T..C..TCC.GC...
HF679405|HDV3 Brazil	G.....G..................T.G........G...T.....TCC.GC...
AB037948|HDV3 Venezuela	.....CG..................TYG.......T.......C..TCC.GC...
AB037947|HDV3 Venezuela	G....CG..................T.G.......T.......C..TCC.GC...
AY648953|HDV4 Taiwan	....TCGA...........................T..............GAG..
AY648952|HDV4 Taiwan	....TCGA...........................T..............GAG..
AF309420|HDV4 Miyako Japan: Miyako	..T..CGA...........C...............AC...T..C..G...GAA..
AB118847|HDV4 Tokyo(JA-T) Japan	....TCGC...........................TC.............GAG..
AB118818|HDV4 Miyako(JA-M2) Japan	.....CGA...........C..T............GA...T..C..G...GAG..
LT604960|HDV5 dFr4824 Mali	.....CG............C......T........TC...T.......CT.....
LT594483|HDV5 dFr7068 Ghana	.....CG............C......T........T....T.......C......
LT594482|HDV5 dFr3634 Cote d Ivoire	.....CG............C......T.......TGA...T.......C......
JX888103|HDV5 Nigeria	.....CG............C......T.......TGA...T.......C......
AM183328|HDV5 dFr2703 Senegal	......G.......C....C..T...T.......TG....T.......C......
MG711710|HDV6 Cameroon	G....CC...............T...T........T..........G.C.GC...
LT604966|HDV6 dFr7029 Cameroon	G...TCC...............T............Y..........G.C.GC...
LT604964|HDV6 dFr3006 Cote d Ivoire	G....AG...............T...T........A..........G.C......
LT594484|HDV6 dFr1594 Angola	.....ACA..................T...................G.C......
AM183332|HDV6 dFr2139 Central African Rep.	.....GCA...........................T..........G.C......
MG711804|HDV7 Cameroon	....TCC...................T........GA...T.....T.C.GA...
MG711773|HDV7 Cameroon	G....CC............................GA...T.......C.GA...
LT604970|HDV7 dFr3363 Cameroon	.....CC............................GA...T.?.....C.GA...
KM110802|HDV7 Cameroon	G.T..C....................T........GA...T.....T.C.GA...
AM183333|HDV7 dFr2158 Cameroon	..T..C....................T........GA...T.......C.GA...
LT604974|HDV8 dFr3111 Rep. of the Congo	..T..C.A..................T.......A...........G...GAT..
LT594488|HDV8 dFr7707 Dem.Rep. of the Congo	..T..C................T...T........GA.........G...GA...
GU177114|HDV8 Gabon	......C...................T........T..........G.C.GAA..
AM183330|HDV8 dFr2072 Senegal	..T..C....................T...................G...GAT..
AM183327|HDV8 dFr2736 Cote d Ivoire	.......A..................T.......AT..........G...GAT..

MK890235|HDV1 Pakistan	C T G C T T C C T C T T G T T C T C G A G G G C C T T C C T T C T T
MH457152|HDV1 Italy	. . . . . . T . . . . A . . . . G .
MH457146|HDV1 Germany	T . . T . . . T . . . . G .
MH457143|HDV1 Spain	. . . . C . . . . T . . . . G .
MG926378|HDV1 Israel	. . . . T . . . . G .
LT604949|HDV1 1063dTn06 Tunisia	. . . . T . . . . G .
LT604940|HDV1 dFr4941 France	. . . . . . T . . . . G .
LT604939|HDV1 dFr4586 Romania	. . . . . . T . . . . G .
KJ744243|HDV1 Iran	T . . . . . . T . . . . T . . . . G .
KJ744237|HDV1 Iran	. . . . T . . . . G .
HQ005364|HDV1 Turkey	T . . . . . . T G . . . . G .
OR669572|HDV Romania	. . . . T . . . . - -
OR669573|HDV Romania	- -
OR669574|HDV Romania	T . . . . . T . . . . - -
OR669575|HDV Romania	. . . . . . T . . . . - - - - - - - - - - - -
OR669576|HDV Romania	. . . . . . T . . . . - -
OR669577|HDV Romania	. . . . T . . . . - -
OR669578|HDV Romania	T . . . . . T . . . . - -
OR669579|HDV Romania	- -
OR669580|HDV Romania	. . . A . . . Y . . . . - -
OR669581|HDV Romania	. . . T C . . . . - -
OR669582|HDV Romania	. . . . C . T . . . . - - - - - - - - - - - -
OR669583|HDV Romania	T . . T C . . T . . . . - - -
OR669584|HDV Romania	T . . T . . T . . . . - -
OR669585|HDV Romania	. . . T . . -
OR669586|HDV Romania	. . . . - - - - - - - - - - -
OR669587|HDV Romania	T . . . . . T . . . . - -
OR669588|HDV Romania	T . . A . . . . - -
OR669589|HDV Romania	. . . . T . . . . - - - - - - - - - - - -
OR669590|HDV Romania	. . . . T . . . . - - - - - - - - - - - -
OR669591|HDV Romania	T . . . . . . T . . . . - -
OR669592|HDV Romania	T . . . . . T . . . . - - - - - - - - - - - -
OR669593|HDV Romania	. . . . . . T . . . . - - - - - - - - - - - -
OR669594|HDV Romania	T . . . . . . T . . . . - - - - - - - - - - -
OR669595|HDV Romania	. . . . - - - - - - - - - - - -
OR669596|HDV Romania	T . . T . . . . - -
OR669597|HDV Romania	. . . . T . . . . - - - - - - - - - - -
OR669598|HDV Romania	T . . . . - -
OR669599|HDV Romania	. . . . T . . . . - -
OR669600|HDV Romania	- -
OR669601|HDV Romania	. . . . T . . . . - - - -
OR669602|HDV Romania	T . . . C . T . . . . - -
OR669603|HDV Romania	. . . . . . T . . . . - - - - - - - - - - -
OR669604|HDV Romania	T . . . . . T . . . . - -
OR669605|HDV Romania	T . . T C . T . . . . - - - - - - - - - - - -
OR669606|HDV Romania	. . . . - -
OR669607|HDV Romania	T . . . C . T . . . . - -
OR669608|HDV Romania	. . . . T . . . . - -
OR669609|HDV Romania	. . . . . . T . . . . - - - - - - - - - - - -
OR669610|HDV Romania	. . . . T . . . . - -
OR669611|HDV Romania	- -
OR669612|HDV Romania	. . . . C . T . . . . - -
OR669613|HDV Romania	T . . Y Y . . T . . . . - - - - - - - - - - - - - - - -
MK234594|HDV2 Taiwan	. . . . . . T . . . . G
LT594481|HDV2 dFr5820 China	. . . . G
KF660598|HDV2 Viet Nam	. . . . G
AJ309879|HDV2 Russia:Yakutia	T . . . . G
AB118846|HDV2 Miyako(JA-M37) Japan	. . . . T . . . . T . . . . G
LT604954|HDV3 dFr6727 Bolivia	. . . . T . . . . C . . . .
HF679406|HDV3 Brazil	T . . . . . . T . . . . C . . . .
HF679405|HDV3 Brazil	T . . . . . . T . . . . C . . . .
AB037948|HDV3 Venezuela	T . . . . . . T . . . .
AB037947|HDV3 Venezuela	T . . . . . . T . . . .
AY648953|HDV4 Taiwan	. . . . T . . . . C . . . .
AY648952|HDV4 Taiwan	. . . . T . . . . C . . . .
AF309420|HDV4 Miyako Japan: Miyako	. . . . . . T T . . . . C . . . .
AB118847|HDV4 Tokyo(JA-T) Japan	. . . . T . . . . C . . . .
AB118818|HDV4 Miyako(JA-M2) Japan	. . . . C . . T . . . . C . . . .
LT604960|HDV5 dFr4824 Mali	T . . . . . . T . . . .
LT594483|HDV5 dFr7068 Ghana	. . . . T . . . .
LT594482|HDV5 dFr3634 Cote d Ivoire	. . . .
JX888103|HDV5 Nigeria	. . . . T . . . . T . . . .
AM183328|HDV5 dFr2703 Senegal	. . . T . . . T . . . .
MG711710|HDV6 Cameroon	T . . . . . . T . . . . - - - - -
LT604966|HDV6 dFr7029 Cameroon	T . . . . . . T . . . . T . . . . G .
LT604964|HDV6 dFr3006 Cote d Ivoire	T . . . . . . T . . . . T . . . . G .
LT594484|HDV6 dFr1594 Angola	T . . . . . . T . . . . T . . . . G .
AM183332|HDV6 dFr2139 Central African Rep.	T . . . . . . T . . . . T . . . . G .
MG711804|HDV7 Cameroon	. . . . T . . . . G .
MG711773|HDV7 Cameroon	. . . . T . . . . - - - - -
LT604970|HDV7 dFr3363 Cameroon	. . . . T . . . . G .
KM110802|HDV7 Cameroon	. . . . T . . . . G .
AM183333|HDV7 dFr2158 Cameroon	. . . . T . . . . G .
LT604974|HDV8 dFr3111 Rep. of the Congo	T . . . . . . T . . . .
LT594488|HDV8 dFr7707 Dem.Rep. of the Congo	T . . . . . . T . . . .
GU177114|HDV8 Gabon	. . . . T . . . .
AM183330|HDV8 dFr2072 Senegal	T . . . . . . T . . . .
AM183327|HDV8 dFr2736 Cote d Ivoire	T . . . . . . T . . . .
